# Nuclear lncRNA HOXD-AS1 suppresses colorectal carcinoma growth and metastasis via inhibiting HOXD3-induced integrin β3 transcriptional activating and MAPK/AKT signalling

**DOI:** 10.1186/s12943-019-0955-9

**Published:** 2019-03-01

**Authors:** Min-Hui Yang, Li Zhao, Lan Wang, Wen Ou-Yang, Sha-Sha Hu, Wen-Lu Li, Mei-Ling Ai, Yi-Qing Wang, Yue Han, Ting-Ting Li, Yan-Qing Ding, Shuang Wang

**Affiliations:** 1 Department of Pathology, Nanfang Hospital, Southern Medical University, Guangzhou, 510515 China; 2 Department of Pathology, School of Basic Medical Sciences, Southern Medical University, Guangzhou, 510515 China; 3 Department of Pathology, The Third Affiliated Hospital of Guangzhou Medical University, Ghuangzhou, 510150 China; 4 The Second Clinical Medical College, Zhujang Hospital, Southern Medical University, Guangzhou, 510515 China

**Keywords:** lncRNA, HOXD-AS1, HOXD3, Colorectal carcinoma, PRC2

## Abstract

**Background:**

Long noncoding RNAs (lncRNAs) have been indicated to play critical roles in cancer development and progression. LncRNA HOXD cluster antisense RNA1 (HOXD-AS1) has recently been found to be dysregulated in several cancers. However, the expression levels, cellular localization, precise function and mechanism of HOXD-AS1 in colorectal carcinoma (CRC) are largely unknown.

**Methods:**

Real-time PCR and in situ hybridization were used to detect the expression of HOXD-AS1 in CRC tissue samples and cell lines. Gain- and loss-of-function experiments were performed to investigate the biological roles of HOXD-AS1 in CRC cell line. RNA pull down, RNA immunoprecipitation and chromatin immunoprecipitation assays were conducted to investigate the mechanisms underlying the functions of HOXD-AS1 in CRC.

**Results:**

We observed that HOXD-AS1 was located in the nucleus of CRC cells and that nuclear HOXD-AS1 was downregulated in most CRC specimens and cell lines. Lower levels of nuclear HOXD-AS1 expression were associated with poor outcomes of CRC patients. HOXD-AS1 downregulation enhanced proliferation and migration of CRC cells in vitro and facilitated CRC tumourigenesis and metastasis in vivo. Mechanistic investigations revealed that HOXD-AS1 could suppress HOXD3 transcription by recruiting PRC2 to induce the accumulation of the repressive marker H3K27me3 at the HOXD3 promoter. Subsequently, HOXD3, as a transcriptional activator, promoted Integrin β3 transcription, thereby activating the MAPK/AKT signalling pathways.

**Conclusion:**

Our results reveal a previously unrecognized HOXD-AS1-HOXD3-Integrin β3 regulatory axis involving in epigenetic and transcriptional regulation constitutes to CRC carcinogenesis and progression.

**Electronic supplementary material:**

The online version of this article (10.1186/s12943-019-0955-9) contains supplementary material, which is available to authorized users.

## Background

Colorectal carcinoma (CRC) is the third most commonly diagnosed cancer in males and the second in females in the world, with approximately 1.3 million new cancer cases and over 0.6 million deaths reported each year [1]. Thus, a better understanding of the molecular mechanisms underlying CRC carcinogenesis and progression is essential for the development of CRC-specific diagnostic markers and novel effective therapies for CRC patients.

Long non-coding RNAs (lncRNAs) consist of a group of RNA molecules that are longer than 200 nucleotides in length with limited protein-coding potential [2]. Accumulating evidence has revealed the dysregulation of lncRNAs expression in a variety of human diseases, including cancer [3, 4]. LncRNA dysregulation contributes to tumour proliferation, metastasis, and recurrence through abnormal regulation of the genes involved in cancer-related cellular processes [5–7]. Thus, understanding the precise molecular mechanisms by which lncRNAs function in various cancers will be critical for exploring new strategies for cancer diagnosis and therapy.

LncRNA HOXD cluster antisense RNA 1 (HOXD-AS1) is the transcript of the HOXD gene cluster located at human chromosome 1q31.2 [8]. Studies have shown that HOXD-AS1 expression was dysregulated in certain tumours, including cervical, ovarian, glioma, liver, bladder, and non-small cell lung cancer (NSCLC) [9–15]. In these cancers, the up-regulation of HOXD-AS1 expression significantly promoted tumour growth, migration and invasion as HOXD-AS1 functioned as a competing endogenous RNA (ceRNA) sponging miRNAs [10–12, 14, 15]. Very recently, a study showed that the increased expression of HOXD-AS1 promotes tumour progression by acting as a ceRNA for miR-217 in CRC [16]. However, this lncRNA is not well-characterized in tumours, especially CRC. The expression levels, cellular localization, precise function and mechanism of HOXD-AS1 in CRC are largely unknown.

Here, we uncovered that HOXD-AS1 located in the nucleus of CRC cells and that HOXD-AS1 expression was downregulated in CRC tissue samples and cell lines. A unique role for HOXD-AS1 in suppressing cell growth, invasion and metastasis was demonstrated by gain- and loss-of-function experiments in vitro and in vivo*.* HOXD-AS1 formed a lncRNA/protein hybrid and mediated the recruitment of Polycomb Repressive Complex (PRC2) to the HOXD3 promoter, a sense gene for HOXD-AS1, leading to significant transcriptional repression of the HOXD3 gene. Then, HOXD3, as a transcription factor, enhanced CRC progression by promoting Integrin β3 transcription and activating the MAPK/AKT signalling pathway. Our work provides novel insights into the function and mechanisms of HOXD-AS1 in CRC progression, which may assist in the development of new therapeutic targets for CRC intervention.

## Methods

### GEO data analysis

Microarray datasets were downloaded from public GEO database (https://www.ncbi.nlm.nih.gov/geo/) and normalized using the robust multichip average (RMA) with R/Bioconductor packages including affy [17].

### Clinical specimens and cell culture

All CRC specimens and matched adjacent non-tumour tissues were obtained with informed consent from Nanfang Hospital, Southern Medical University (Guangzhou, China). Freshly frozen tumor samples from 35 CRC patients were selected for real-time PCR. 164 paraffin-embedded CRC samples were used for immunohistochemistry (IHC) and 150 samples for in situ hybridization (ISH). Complete follow-up, ranging from 1 to 117 months, and no patient received any pre-operative chemotherapy and radiotherapy.

The human CRC cell lines SW620, SW480, DLD-1, HCT116, and LoVo, normal colon epithelial cell line FHC, and the human embryonic kidney 293 T cell lines were obtained from American Type Culture Collection. M5 cell line, a subclone with enhanced metastasis ability, was derived from SW480 through in vivo selection in our laboratory. All of the CRC and FHC cell lines were cultured in PRMI 1640 medium (Gibco, USA), HEK-293 T was cultured in Dulbecco’s modified Eagle’s medium (DMEM; Gibco, USA). All the medium was added with 10% FBS (Gibco, USA). All the cells were cultured at 37 °C with 5% CO_2_.

### Real-time PCR, in situ hybridization, RNA fluorescence in situ hybridization, Western blot, and immunohistochemistry

Real-time PCR, in situ hybridization (ISH), fluorescence in situ hybridization (FISH), western blot and immunohistochemistry (IHC) were conducted according to previously described methods [18]. Details are provided in (Additional file 1: Supplementary Materials and Methods).

### Construction of CRC cell lines with stably overexpressed HOXD-AS1 and HOXD3

To evaluate the expression of HOXD-AS1, the HOXD-AS1 sequence was synthesized and subcloned into a pcDNA3.1 vector (GenePharma, China). CRC cell lines with ectopic expression of HOXD-AS1 were achieved by stably transfecting pcDNA3.1-HOXD-AS1 and CRC cells transfected with empty pcDNA vector were used as control. The cDNA encoding the CDS of HOXD3 was amplified by PCR and subcloned into the Kpn I and Not I sites of the pcDNA3.0 vector. The expression of HOXD-AS1 and HOXD3 were detected by real-time PCR.

### Construction of cell lines with stably downregulated HOXD-AS1 and HOXD3

There shRNA sequence specially targeting HOXD-AS1 or HOXD3 were designed and synthesized, and clone into a pGU6/GFP/Neo-shRNA vector (GenePharma, China). The most effective shRNA sequence in achieving knockdown of HOXD-AS1 or HOXD3 expression was selected to be constructed into lentiviruses by GenePharma (China). Lentiviruse production and infection were performed as previously described [18]. Details are provided in (Additional file 1: Supplementary Materials and Methods).

### Cell in vitro proliferation, flow cytometry cell cycle, colony formation, wound-healing, invasion assays *and* in vivo tumorigenic and metastasis assays

Cell in vitro proliferation, flow cytometry cell cycle, colony formation, wound-healing, invasion assays and in vivo tumorigenic and metastasis assays were performed according to previously described methods [18]. Further details are provided in (Additional file 1: Supplementary Materials and Methods section).

### RNA pull-down assay

HOXD-AS1 and its antisense RNA was in vitro transcribed from the vector pcDNA3.1-HOXD-AS1 using T7 RNA polymerase (Roche Diagnostics, USA), and then treated with DNase I, and subsequently purified with an MEGAscript™ T7 Transcription Kit (Invitrogen, CA). Next, HOXD-AS1 and its antisense RNA were biotin-labeled with the Biotin RNA Labeling Mix (Roche Diagnostics, USA). The nuclear lysates of SW620 were incubated with biotinylated RNAs and magnetic beads (Life Technologies, USA) and subsequently washed. The proteins present in the pull-down material were resolved by SDS-PAGE, and then silver-stained. Special bands were excised and analyzed by qualitative mass spectrometry in a firm (Wininnovate Bio, China).

### RNA immunopreciptiation

RNA immunopreciptiation (RIP) assay was performed using Magna RIP RNA-Binding Protein Immunoprecipitation Kit (Millipore, MA) following the manufacturer’s instructions. Briefly, cells were crosslinked with 1% (*w*/*v*) formaldehyde and lysed in complete RIP lysis buffer, and subsequently incubated with magnetic beads conjugated with anti-SUZ12- and anti-EZH2-antibodies overnight at 4 °C. RNA purified from the RNA-protein complex was amplified and detected by real-time PCR.

### Chromatin immunopreciptiation

Chromatin immunopreciptiation (ChIP) assays were performed using EZ-Magna ChIP™ A/G kit (Millipore, USA) with anti-H3K27me3-antibody (rabbit monoclonal, #9733, CST, USA), anti-SUZ12-antibody (rabbit polyclonal, ab12073, Abcam, UK), anti-EZH2-antibody (rabbit monoclonal, #5246, CST, USA), or anti-HOXD3-antibody (rabbit polyclonal, ab22840, Abcam, UK). Normal mouse immunoglobulin G (IgG) was used as a negative control. The immunoprecipitated DNA was purified, and quantified by real-time PCR with special primers. The primers were shown in (Additional file 2: Table S1).

### Duel-luciferase reporter assay

The wide type and the mutation of the promoter segment sequences of Integrin β3 were synthesized and inserted into a pGL3-basic vector (Promega, USA) respectively. HEK-293 T cells and SW620 cells were respectively plated in 24-wall plates at 5 × 10^3^ cells per well 8 h before transfection. The cells were co-transfected with a mixture of 500 ng pGL3-basic-ITBG3 promoter, 5 ng Renilla and 500 ng pcDNA3.0-HOXD3 or control. 48 h later, the luciferase activity was measured using the Dual-Luciferase Reporter Assay System (Promega, USA).

### Statistical analysis

All statistical analyses were performed using the SPSS 19.0 software (SPSS, USA). Student’s t-test (two tailed), one-way ANOV, were used to calculate the difference between two groups or more than two groups. *χ*^*2*^ test was used to estimate the correlation between the expression and clinicopathologic features. Cumulative survival was estimated using the Kaplan–Meier method (the log-rank test). The multivariate survival analysis was performed using Cox regression model. A probability value of 0.05 or less was considered as significance.

## Results

### Nuclear HOXD-AS1 expression is reduced in CRC and negatively associated with poor prognosis in CRC patients

**Fig. 1:**
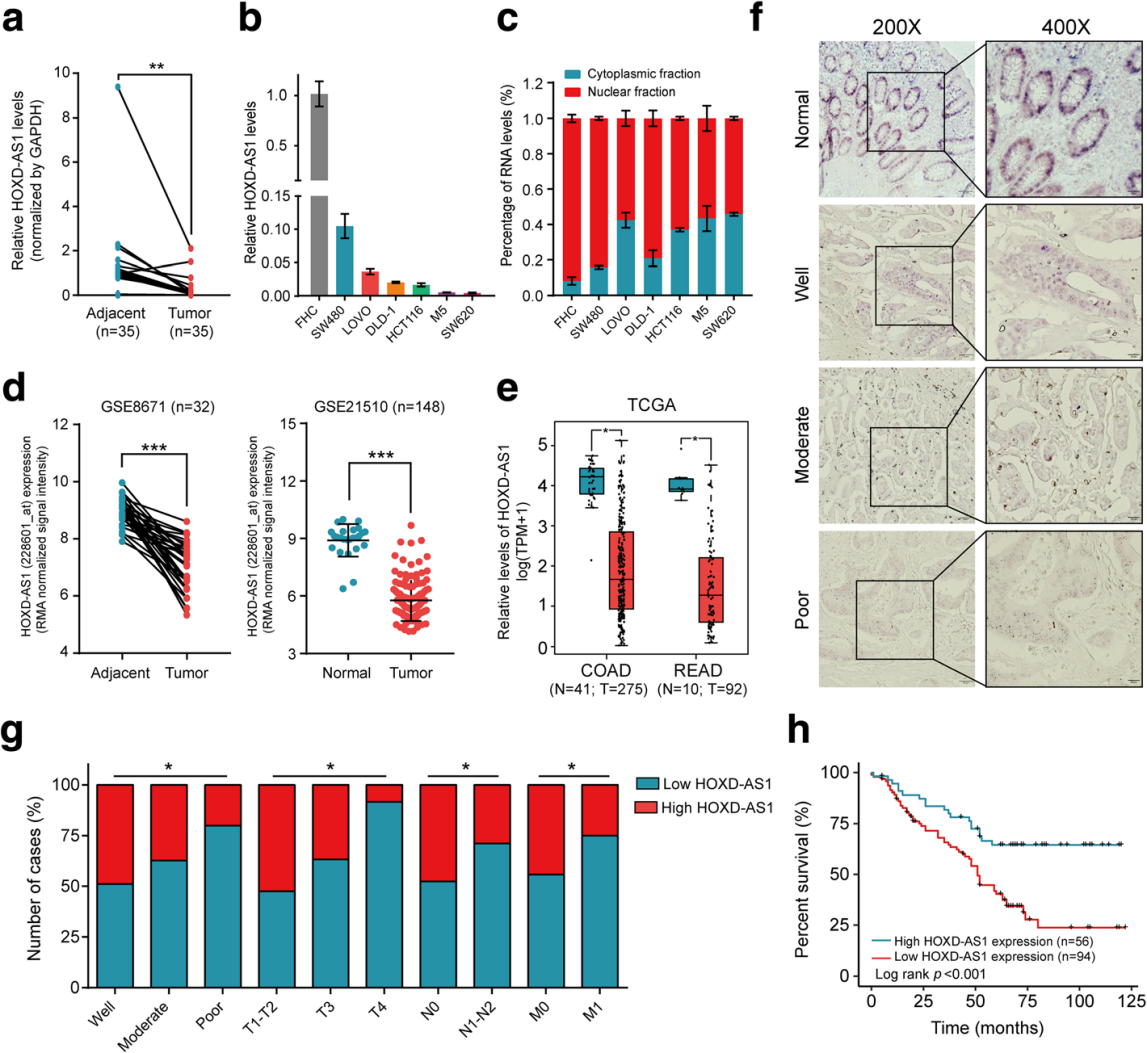
The down-regulation of nuclear HOXD-AS1 predicts poor outcome in CRC patients. **a** Expression levels of HOXD-AS1 in 35 paired CRC and adjacent non-cancerous tissues. **b** Expression levels of HOXD-AS1 in CRC and colon mucosa epithelial (FHC) cell lines. **c** The proportion of HOXD-AS1 in the cytoplasmic and nuclear fraction in CRC and FHC cells were detected by real-time PCR. HOXD-AS1 expression was normalized to GAPDH (exported to cytoplasm) and U6 (nuclear retained). **d-e** The analysis of HOXD-AS1 expression in CRC compared with normal tissues in CRC microarray profile (GES8671, Paired t test; GSE21510, Mann-Whitney test) and RNA-seq in TCGA database. **f** Expression analysis of HOXD-AS1 in normal colorectal mucosa and CRC tissues by ISH. Scale bars, 50 μm (200×) or 20 μm (400×), were shown in the right corner of each picture. **g** Graphical illustration of statistical HOXD-AS1 distribution in CRC patients. **h** Kaplan–Meier analysis of overall survival in all patients with CRC according to HOXD-AS1 expression. (log-rank test). For a-c, data are presented as means ± SD in three independent experiments. *P < 0.05, **P < 0.01, ***P < 0.001

To investigate the role of HOXD-AS1 in CRC, we first assessed HOXD-AS1 expression levels in 35 paired primary CRC tissue and matched adjacent nontumor tissue samples by real-time PCR. The results showed that the expression of HOXD-AS1 was significantly reduced in the CRC tissue samples (*P* = 0.007, Fig. 1a). Moreover, HOXD-AS1 expression was down-regulated in all the CRC cell lines compared with a normal cell line (FHC) cells (*P* = 0.0012, Fig. 1b). Furthermore, nuclear and cytoplasmic RNA fractions were prepared from CRC and FHC cells to observe the subcellular localization of HOXD-AS1. As shown in Fig. 1c, we found that HOXD-AS1 was mainly enriched in the nucleus (*P* < 0.001, Fig. 1c). To further explore the expression pattern of HOXD-AS1 in CRC, we also performed an integrative analysis of HOXD-AS1 expression in a public microarray profile dataset from the Gene Expression Omnibus (GEO) database and the Cancer Genome Atlas (TCGA). Consistent with our above results, HOXD-AS1 expression was downregulated in CRC tissue (GSE8671 [19], GSE21510 [20], GSE32323 [21], GSE41328 [22], GSE23878 [23], GSE9348 [24] and TCGA (Fig. 1d and e, and Additional file 3: Figure S1).

In addition, ISH was performed to determine the quantity and sites of HOXD-AS1 expression in an independent cohort of 150 paraffin-embedded CRC samples. We observed that HOXD-AS1 was predominantly expressed in the cell nucleus (Fig. 1f) and that HOXD-AS1 was expressed in only 37.3% (56/150) of the CRC samples but in 76.92% (30/39) of the adjacent noncancerous tissue samples. The levels of HOXD-AS1 were significantly downregulated in CRC tissue (*P* < 0.001). Taken together, these findings uncovered that HOXD-AS1 expression is downregulated in CRC, which indicated that HOXD-AS1 is a suppressor in CRC.

Then we divided all CRC patients into high and low HOXD-AS1 expression level groups according to the median value. The results showed that lower expression of HOXD-AS1 was significantly correlated with poor differentiation (*P* = 0.047), advanced TNM stage (*P* < 0.05, Fig. 1g) and poorer survival in CRC patients (*P* < 0.05, Fig. 1h and Table 1). In addition, multivariate Cox regression analyses showed that HOXD-AS1 expression was an independent prognostic factor for the outcomes of patients with CRC (Additional file 2: Table S2).

**Table 1:** Correlation between the clinicopathological features and expression of HOXD-AS1

clinicopathological variables	N	high(%)	low(%)	χ2	*P* value
All cases	150	56	94		
Age					
< mean (56)	71	24 (33.8)	47 (66.2)	0.718	0.405
≥ mean (56)	79	32 (40.5)	47 (59.5)		
Gender					
Male	89	33 (37.1)	56 (62.9)		
Female	61	23 (37.7)	38 (62.3)	0.006	1
Tumor Size					
< 5 cm	96	40 (41.7)	56 (58.3)	2.14	0.162
≥ 5 cm	54	16 (29.6)	38 (70.4)		
Tumor Differentiation					
Well	45	22 (48.9)	23 (51.1)	6.121	0.047
Moderate	75	28 (37.3)	47 (62.7)		
Poor	30	6 (20.0)	24 (80.0)		
T classification					
T1-T2	40	19 (47.5)	21 (52.5)	6.096	0.047
T3	98	36 (36.7)	62 (63.3)		
T4	12	1 (8.3)	11 (91.7)		
N classification					
N0	85	38 (44.7)	47 (55.3)	4.557	0.033
N1-N2	65	18 (27.7)	47 (72.3)		
M classification					
M0	113	48 (42.5)	65 (57.5)	4.637	0.031
M1	37	8 (21.6)	29 (78.4)		

### HOXD-AS1 suppresses CRC cell proliferation, cell-cycle progression, migration and invasion in vitro

**Fig. 2:**
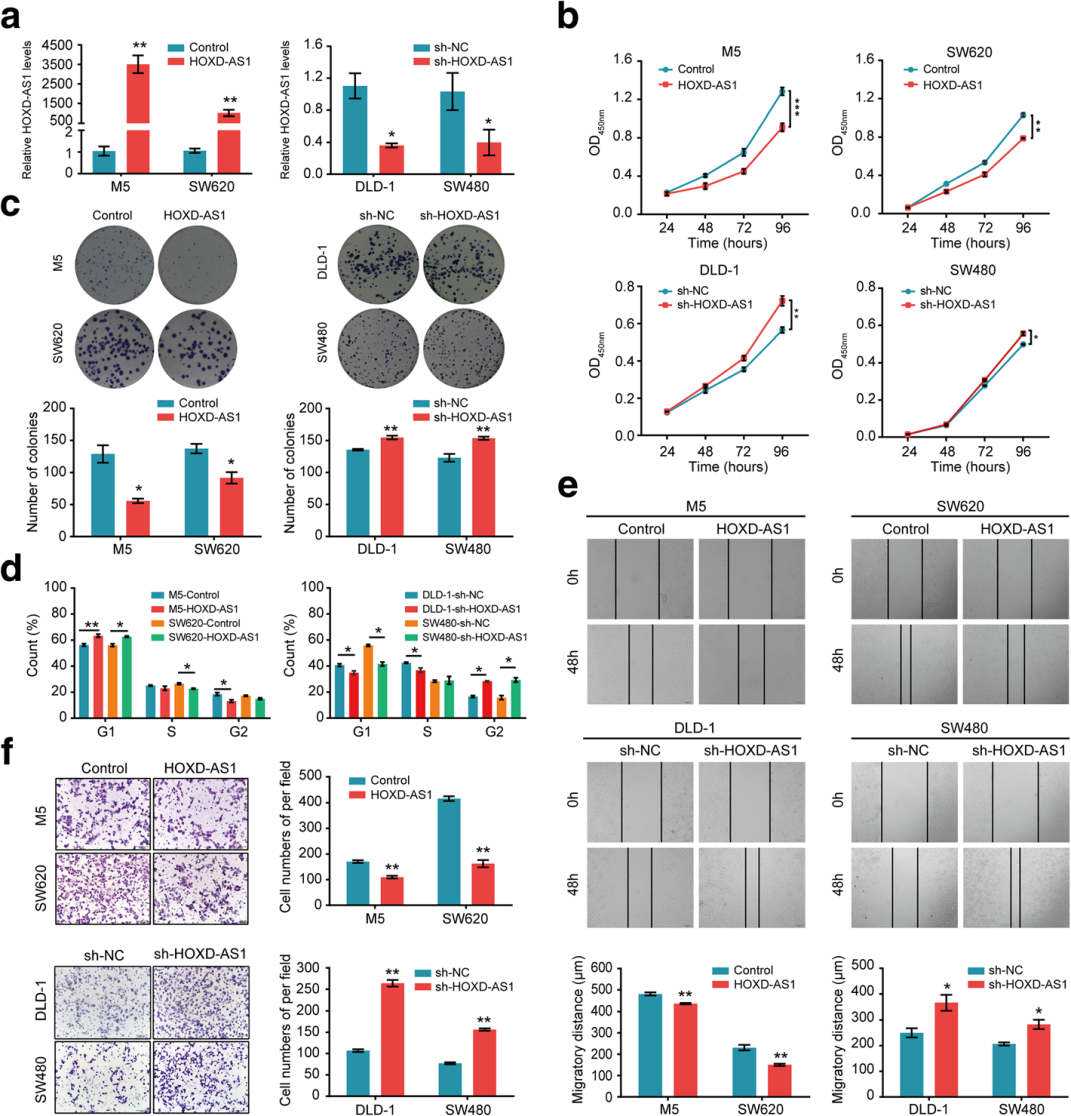
HOXD-AS1 suppresses CRC cell proliferation, cell-cycle progression, migration and invasion in vitro. **a** HOXD-AS1-overexpressing SW620 and M5 cell lines were established by the transfection of pcDNA3.1-HOXD-AS1. And infecting SW480 and DLD-1cells with a lentivirus vector harbouring shRNA-HOXD-AS1 to knocked down the endogenous expression of HOXD-AS1 in cells. HOXD-AS1 levels in cells were detected by real-time PCR. **b** CCK-8 assays were performed to determine the proliferation of HOXD-AS1-overexpressed or -depleted CRC cells. **c** Colony-forming assays were performed to determine the effects of HOXD-AS1-overexpression or -depletion on the growth of CRC cells. The diameter > 50 cells was scored. **d** Cell cycle progression was analyzed by flow cytometry. **e** The migration potencies of CRC cells with the indicated treatments were detected by using wound healing assay. **f** Invasion assays were used to determine the effects of HOXD1-overexpression or -depletion on the invasion ability of CRC cells. For a-f, data were expressed as means ± SD in three independent experiments. *P < 0.05, **P < 0.01, ***P < 0.001

Two HOXD-AS1-overexpressing CRC cell lines were established by the transfection of pcDNA3.1-HOXD-AS1. In contrast, we knocked down the endogenous expression of HOXD-AS1 in SW480 and DLD-1 cells by infecting the cells with a lentivirus vector harbouring shRNA-HOXD-AS1. Real-time PCR analysis confirmed that HOXD-AS1 expression was successfully up-regulated or down-regulated in the corresponding CRC cells (Fig. 2a). A significantly slower proliferation rate was observed in HOXD-AS1-overexpressing SW620 and M5 cells compared with control cells (Fig. 2b). Moreover, a colony formation assay further confirmed that the up-regulation of HOXD-AS1 expression contributed to the repression of colony formation by CRC cells (Fig. 2c). Meanwhile, Flow cytometry assays demonstrated that HOXD-AS1 overexpression could increase the percentage of G1-phase cells in both SW620 and M5 cells (*P* < 0.05, Fig. 2d). In contrast, the depletion of HOXD-AS1 promoted proliferation, cell-cycle progression and colony formation in CRC cells (Fig. 2b-d).

Next, we performed a scratch-wound healing assay and a Transwell assay to determine the effects of HOXD-AS1 on CRC cell migration and invasion. As shown in Fig. 2e and f, after HOXD-AS1 overexpression, the migratory and invasion abilities of SW620 and M5 cells were both remarkably impaired, while the knockdown of HOXD-AS1 expression dramatically increased cell migration and invasion.

### HOXD-AS1 inversely regulated the expression of HOXD3, a neighbouring gene

**Fig. 3:**
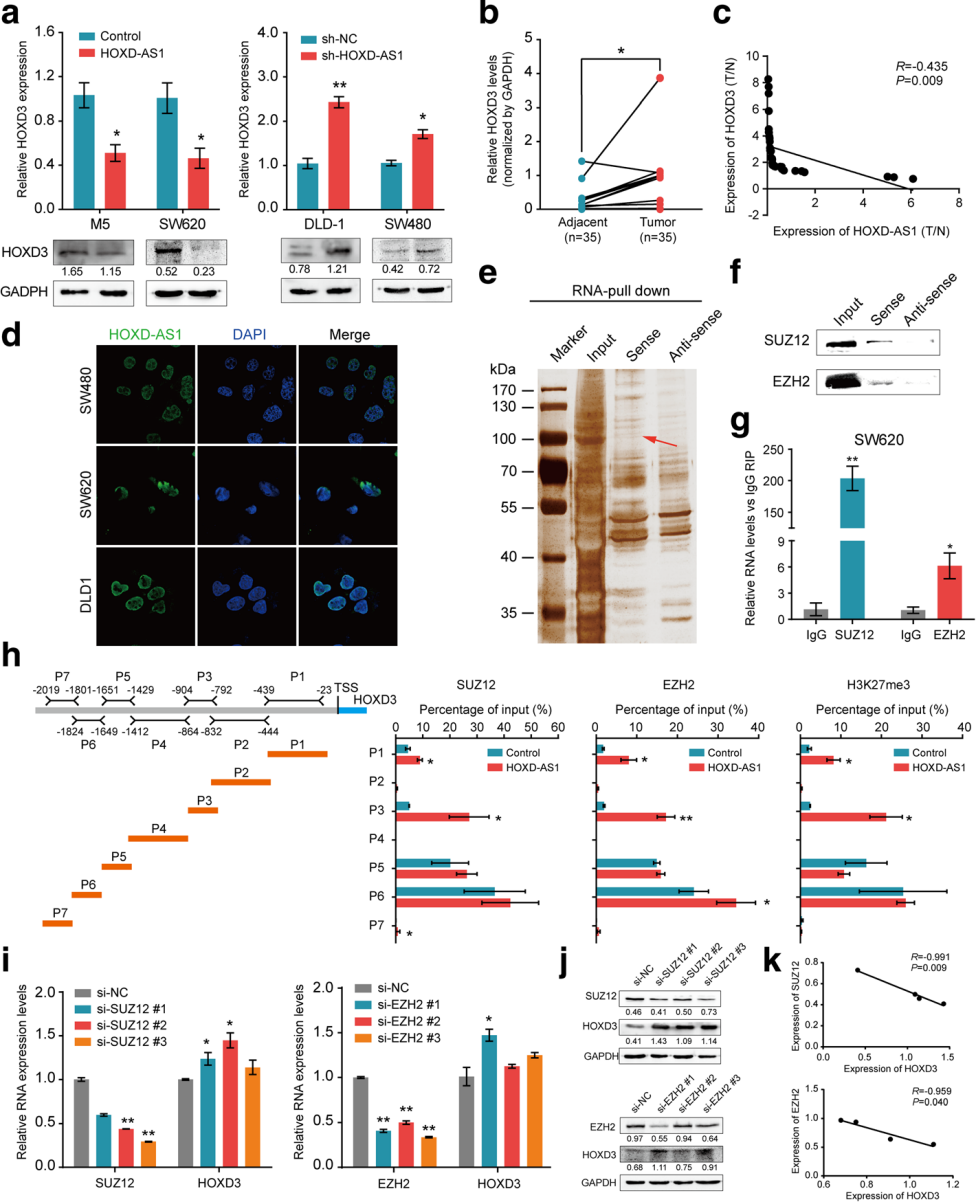
HOXD-AS1 inversely regulates the expression of neighbouring gene HOXD3. **a** Expression of HOXD3 in CRC cells were detected by real-time PCR (upper) and Western blot (down) when HOXD-AS1-overexpression or -depletion, respectively. **b** Expression levels of HOXD-AS1 in 35 paired CRC and adjacent non-cancerous tissues. **c** The expression correlation between HOXD-AS1 and HOXD3 was detected by real-time PCR in 35 paired CRC and adjacent non-cancerous tissues. **d** FISH analysis of the subcellular location of HOXD-AS1 in CRC cells. HOXD-AS1 mainly enriches in nucleus. **e** To identify the proteins associated with HOXD-AS1 by RNA pull-down assays. Biotinylated HOXD-AS1 and antisense RNA were incubated with cell extracts, and the associated proteins were resolved by SDS-PAGE. The HOXD-AS1-sense-special bands (arrows) in SW620 nuclear lysates group were excised and analyzed by mass spectrometry. **f** Western blot was used to detect SUZ12 and EZH2 in pull-down products. **g** RIP assays were performed in SW620 cells using anti-SUZ12, anti-EZH2 or nonspecific IgG antibodies respectively. Real-time PCR was performed to determine amount of RNA associated with SUZ12, EZH2 or IgG compared with the input control. **h** ChIP assays were performed in HOXD-AS1 overexpressed(SW620-HOXD-AS1)and control cells using anti-EZH2-, anti-SUZ12-, and anti-H3K27me3-antibodies or IgG antibody respectively. To determine the specific binding site of PRC2 complex and the promoter region of HOXD3, we divided HOXD3 promoter region into 7 segments. The enrichments of 7 segments of HOXD3 gene promoter DNA associated with antibodies were examined by real-time PCR. **i-j** The expression of HOXD3 were detected by **i** real-time PCR and **j** Western blot in SUZ12 or EZH2-depleted SW620 and control cells, respectively. **k** The quantitative data analysis of **j** to show the expression pattern between EZH2 or SUZ12 and HOXD3, respectively. For a, b, c, g, h, i and k, data were presented as means ± SD in three independent experiments. *P < 0.05, **P < 0.01

There is increasing evidence that antisense transcript lncRNAs can directly or indirectly regulate the expression of their neighbour genes [25]. We searched for the genes adjacent to HOXD-AS1 in the UCSC database (http://genome.ucsc.edu/) and found that HOXD-AS1 is located between the HOXD family members HOXD1 and HOXD3 (Additional file 4: Figure S2a). We next examined whether HOXD-AS1 regulates its neighbouring genes. When the expression levels of HOXD-AS1 were increased, HOXD3 mRNA expression was significantly decreased in both SW620 and M5 cells (Fig. 3a). However, we did not detect any obvious change in the expression of HOXD1, another sense-cognate gene for HOXD-AS1 (Additional file 4: Figure S2b). In parallel, western blot results showed that the HOXD3 protein expression was reduced in HOXD-AS1-overexpressing CRC cells compared with control cells (Fig. 3a). In contrast, cells treated with a specific siRNA that knocked down HOXD-AS1 expression showed significantly elevated HOXD3 expression compared with cells treated with a negative control siRNA (Fig. 3a). These results confirmed the regulation of HOXD3 expression by HOXD-AS1 in vitro at the mRNA and protein levels.

Next, we measured HOXD3 mRNA levels in the same set of CRC tissues and cells shown in Fig. 1a and b. Interestingly, in contrast to the HOXD-AS1 expression pattern, HOXD3 expression was elevated in the majority of the CRC samples and all CRC cell lines (*P* < 0.05, Fig. 3b and Additional file 5: Figure S3a). An inverse correlation between HOXD-AS1 and HOXD3 expression was observed in our tissue samples (*R* = 0.435, *P =* 0.009, Fig. 3c). These results suggest that HOXD-AS1 can inversely regulate HOXD3, but not HOXD1, at the transcriptional level.

### Accumulation of histone H3K27 trimethylation of the HOXD3 promoter is dependent on the level of HOXD-AS1 recruiting PRC2

Since HOXD-AS1 was determined to enrich in nucleus of CRC cells by RNA fraction assays (Fig. 1c), a similar subcellular location for HOXD-AS1 in CRC cells was confirmed by FISH (Fig. 3d), which is consistent with a potential role for HOXD-AS1 in epigenetic or transcriptional regulation.

Then, we performed an RNA pull-down assay with the nuclear fractions to identify the protein interactions of HOXD-AS1. The bands specific to HOXD-AS1 were subjected to mass spectrometry. Interestingly, two PRC2 subunits, suppressor of Zeste 12 Homologue (SUZ12) and Enhancer of Zeste Homologue 2 (EZH2), were the specific proteins associated with HOXD-AS1 by mass spectrometry (Fig. 3e and Additional file 2: Table S3). Using antibodies specific to SUZ12 and EZH2, we observed that they were enriched in the HOXD-AS1 pull down proteins (Fig. 3f). Meanwhile, RIP assays with antibodies against SUZ12 and EZH2 were carried out to further demonstrate a significant enrichment of HOXD-AS1 in the anti-SUZ12- or anti-EZH2-antibody pull downs compared with the IgG antibody pull down, a nonspecific control (Fig. 3g and Additional file 6: Figure S4a).

Next, we determined whether the PRC2 complex was bound to the promoter region of HOXD3 and whether the over-expression of HOXD-AS1 affected PRC2 occupancy in this region. We observed a significant enrichment of HOXD3 promoter DNA in ChIP assays using anti-SUZ12- and anti-EZH2-antibodies. The ability of the PRC2 complex to bind to the HOXD3 promoter was significantly increased in the CRC cells overexpressing HOXD-AS1 compared with the control cells expressing endogenous levels of HOXD-AS1 (Fig. 3h and Additional file 6: Figure S4b), suggesting that anti-SUZ12- and anti-EZH2-antibodies can both bind to the HOXD3 promoter region. These results indicate a correlation between the higher levels of HOXD-AS1 and the higher PRC2 occupancy of the HOXD3 promoter.

The PRC2 complex can silence gene expression by inducing trimethylation of histone 3 lysine 27 (H3K27me3), and we reasoned that it may act to regulate the levels of H3K27me3 in the HOXD3 promoter region. Interestingly, there was a significant enrichment of HOXD3 gene promoter DNA, especially the P1 (− 439〜-23 bp) and P3 (− 904〜-792 bp) regions, associated with H3K27me3 when HOXD-AS1 was overexpressed in CRC cells. Meanwhile, we also determined that the depletion of both EZH2 and SUZ12 mediated by siRNA targeting EZH2 or SUZ12, respectively, resulted in increased expression of HOXD3 (Fig. 3i and j). The expression levels of HOXD3 and EZH2 were negatively correlated in these sample (*R* = 0.991, *P* = 0.009, Fig. 3k upper). A similar correlation was found between HOXD3 and SUZ12 expression (*R* = 0.959, *P* = 0.040, Fig. 3k down). Taken together, these results indicated that the H3K27me3 levels at the HOXD3 promoter are dependent on the amount of HOXD-AS1 recruiting PRC2.

### The up-regulation of HOXD3 expression predicts poor outcome in CRC patients and promotes CRC progression

**Fig. 4:**
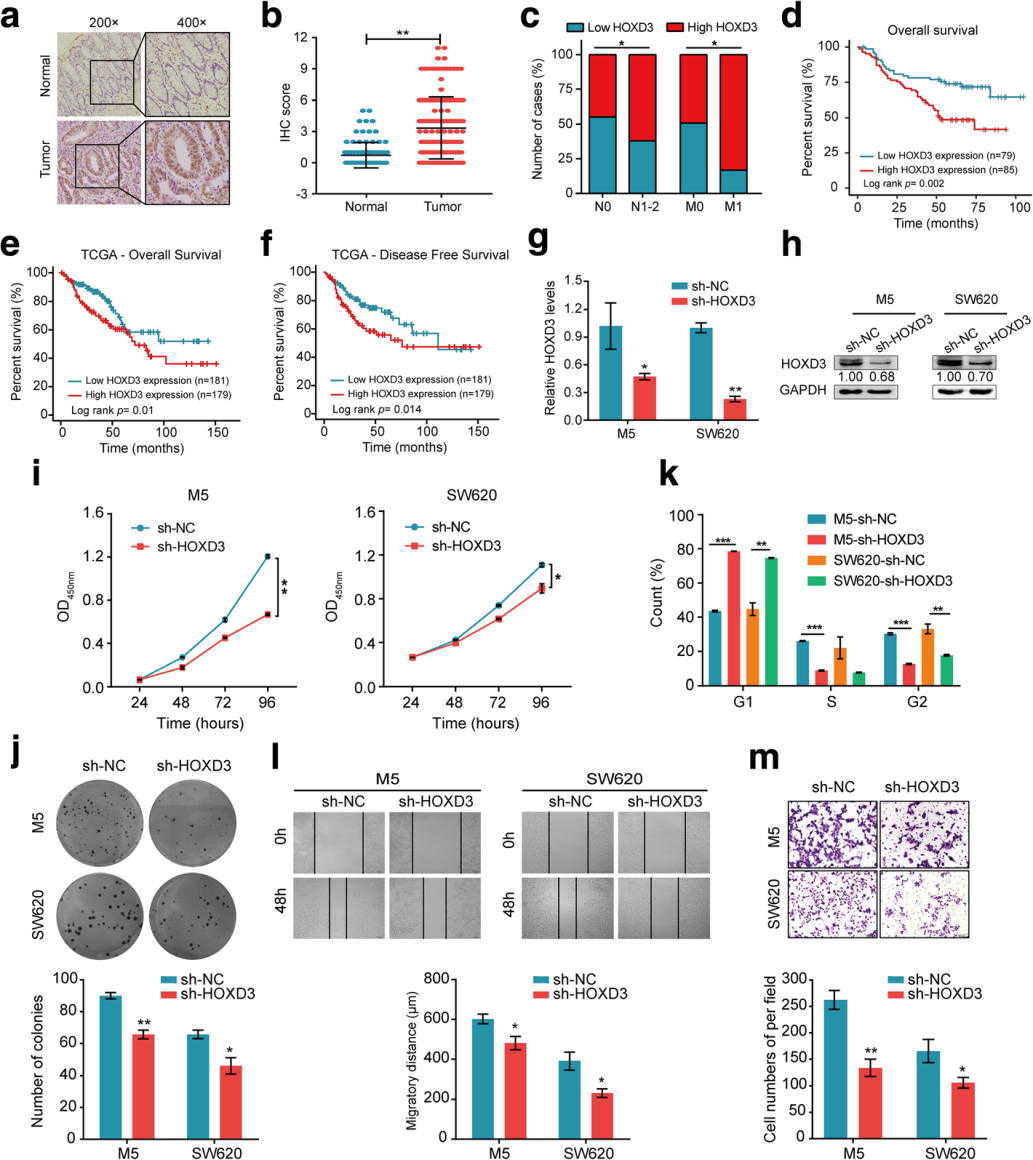
The up-regulation of HOXD3 expression predicts poor outcome in CRC patients and promotes CRC progression. **a-b** Expression analysis of HOXD-AS1 in normal colorectal mucosa and CRC tissues by **a** IHC and **b** the statistical analysis for HOXD3 expression. **c** Graphical illustration of statistical HOXD3 distribution in CRC patients. **d-f** Kaplan–Meier analysis of overall survival in all patients with CRC according to HOXD3 expression in clinical samples (overall survival, *n* = 164, log-rank test) and TCGA database (overall survival and disease-free survival, *n* = 360, log-rank test). **g** Infecting M5 and SW620 cells with a lentivirus vector harbouring shRNA-HOXD3 to knocked down the endogenous expression of HOXD3 in cells. HOXD3 levels in cells were detected by real-time PCR. **h** HOXD3 levels in cells were detected by Western blot. **i** CCK-8 assays were performed to determine the proliferation of HOXD3- depleted CRC cells. **j** Colony-forming assays were performed to determine the effects of HOXD3 depletion on the growth of CRC cells. The diameter > 50 cells was scored. **k** Cell cycle progression was analyzed by flow cytometry. **l** The migration potencies of CRC cells with the indicated treatments were detected by using wound healing assay. **m** Invasion assays were used to determine the effects of HOXD3 depletion on the invasion ability of CRC cells. For b, g, i, j, k, l and m, data are presented as means ± SD in three independent experiments. *P < 0.05, **P < 0.01, ***P < 0.001

HOXD3, a member of the HOX transcription factor gene family, has been reported to be aberrantly expressed in some cancers [26–28]. To investigate the role of HOXD3 in CRC, we detected the expression levels of HOXD3 protein in 164 paraffin-embedded CRC tissue samples by IHC. The results showed that the CRC samples displayed higher expression of HOXD3 than the noncancerous samples (*P* = 0.046, Fig. 4a and b). Analyses of the results indicated that higher expression of HOXD3 was significantly correlated with more advanced N stage (*P* = 0.038), more advanced M stage (*P* = 0.033) in CRC patients (Fig. 4c and Table 2). Consistent with the above results, a Kaplan-Meier analysis also showed a positive correlation between tumour-associated HOXD3 expression and poor overall survival (*P* = 0.01, Fig. 4d) or overall survival (*P* = 0.01, Fig. 4e) and disease-free survival (*P* = 0.014, Fig. 4f) in CRC patients in the TCGA cohort. Univariate and multivariate Cox regression analyses showed that HOXD3 expression was an independent prognostic factor for the outcomes of CRC patients (Additional file 2: Table S4).

**Table 2:** Correlation between the clinicopathological features and expression of HOXD3

clinicopathological variables	N	low(%)	high(%)	χ2	*P* value
All cases	164	79	85		
Gender					
male	89	41 (46.1)	48 (53.9)	0.345	0.638
female	75	38 (50.7)	37 (49.3)		
Age(years)					
< 56	72	37 (51.4)	35 (48.6)	0.532	0.53
≥ 56	92	42 (45.7)	50 (54.3)		
Tumor size(cm)					
**< 5**	96	45 (46.9)	51 (53.1)	0.156	0.752
**≥ 5**	68	34 (50.0)	34 (50.0)		
Tumor differentiation					
Good	56	29 (51.8)	27 (48.2)	1.447	0.485
Moderate	77	38 (49.4)	39 (50.6)		
Poor	31	12 (38.7)	19 (61.3)		
T-stage					
T1-T2	31	20 (64.5)	11 (35.5)	4.397	0.111
T3	127	57 (44.9)	70 (55.1)		
T4	6	2 (33.3)	4 (66.7)		
N-stage					
0	98	54 (55.1)	44 (44.9)	4.686	0.038
1–2	66	25 (37.9)	41 (62.1)		
M-stage					
0	152	77 (50.7)	75 (49.3)	5.147	0.033
1	12	2 (16.7)	10 (83.3)		

To further investigate the biological roles of HOXD3, we also performed loss- and gain-of-function experiments in CRC cells by siRNA-mediated knockdown (Fig. 4g and h) or ectopic overexpression (Additional file 5: Figure S3) of HOXD3. We found that the stable knockdown of HOXD3 expression caused a significant reduction in cell proliferation, as determined by CCK-8, colony formation and cell-cycle progression assays (Fig. 4h-k). Meanwhile, we also observed that HOXD3 ectopic overexpression increased the proliferative abilities of HCT116 and DLD1 cells (Additional file 5: Figure S3c-3e), similar to the effects of HOXD-AS1 depletion. Next, we determined whether HOXD3 affected CRC migration and invasion. We observed that the downregulation of HOXD3 expression resulted in a remarkable suppression of the motility and invasiveness of M5 and SW620 cells (Fig. 4l and m). In contrast, the up-regulation of HOXD3 expression promoted HCT116 and DLD1 cell migration and invasion (Additional file 5: Figure S3f and 3g). Taken together, these results suggest that HOXD3 should be a prognostic biomarker for CRC and promotes CRC cell proliferation and migration in vitro as an oncogene.

### Inhibitory effects of HOXD-AS1 on CRC progression require HOXD3

To test the contribution of HOXD3 to the HOXD-AS1-mediated decrease in CRC cell proliferation and migration, we performed rescue experiments. We up-regulated the level of HOXD3 expression in HOXD-AS1-overexpressing SW620 cells by transfecting the pcDNA3.1-HOXD3 vector. The expression levels of HOXD-AS1 and HOXD3 were confirmed by real-time PCR (Additional file 7: Figure S5a). We first carried out functional experiments in vitro and found that the ectopic expression of HOXD3 could significantly reduce the repressive effects on CRC cell proliferation, colony formation, cell-cycle progression, migration and invasion mediated by HOXD-AS1 in vitro by CCK8, colony formation, scratch-wound healing and Transwell assays, respectively (Additional file 7: Figure S5b-5f).

**Fig. 5:**
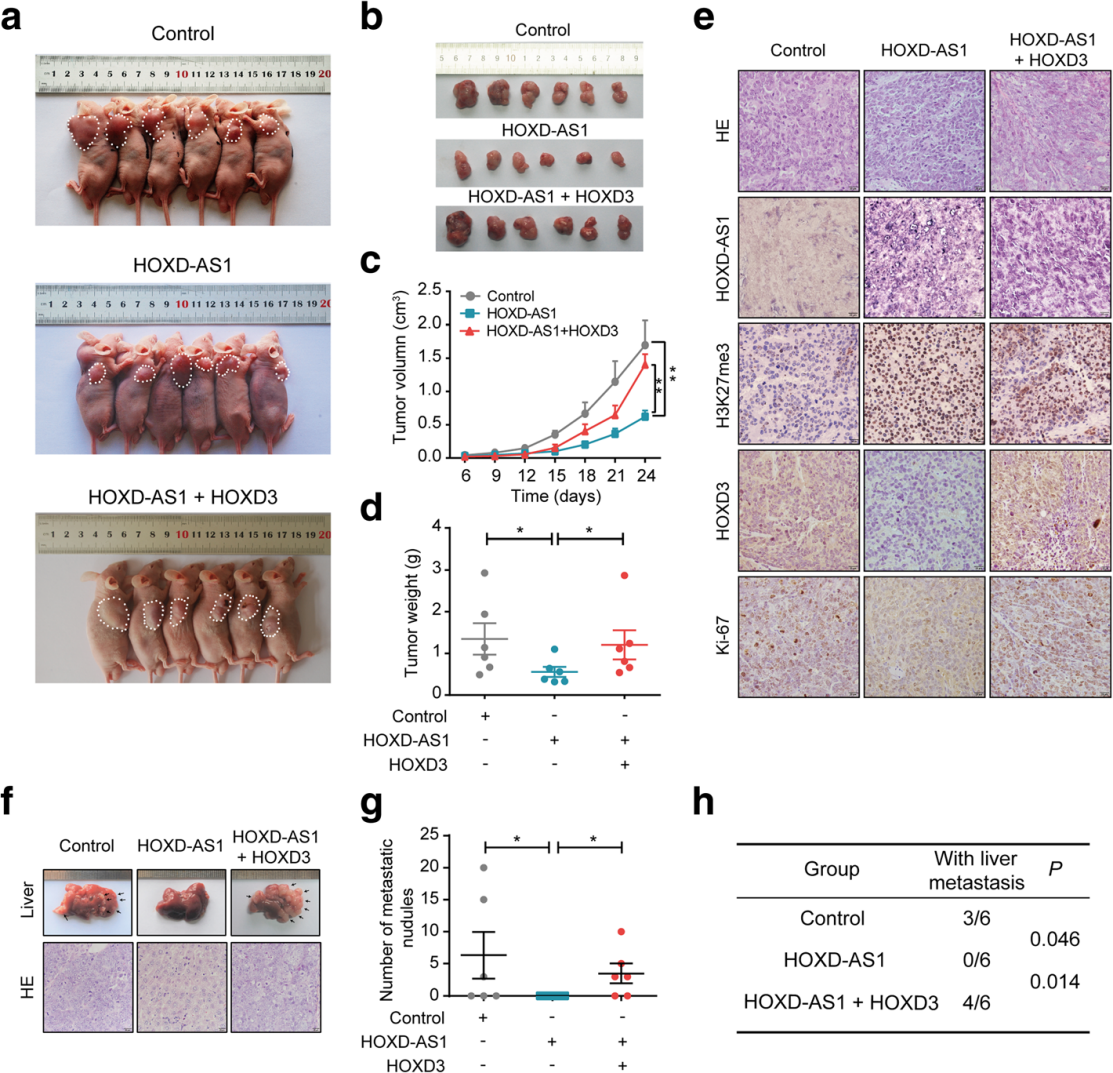
HOXD3 is required for the HOXD-AS1-mediated progress of CRC in vivo. **a**–**d** in vivo tumorigenesis experiment in nude mice. **a** These graphs show the tumor xenografts 24 days after ectopic-subcutaneous implantation in nude mice with SW620-HOXD-AS1, SW620-HOXD-AS1 + HOXD3 and control cells. **b** The gross of xenografts. **c** The effect of HOXD-AS1 or HOXD3 on CRC tumor growth was evaluated based on tumor volume in the three groups. **d** The statistic results of final tumor weights. **e** The images of H&E staining, ISH for HOXD-AS1 and IHC for HOXD3, H3K27me3 and Ki-67 in xenografts were shown. **f**–**h** Intrasplenic injections to establish liver metastasis model in nude mice. **f** These graphs show SW620-HOXD-AS1, SW620-HOXD-AS1 + HOXD3 and control cells metastasis in vivo*.* 6 weeks after CRC cells’ Intrasplenic injections. Liver metastases were showed (upper) and the tissues were stained by H&E staining (down). **g** The statistical analysis of number of liver metastatic nodules and **h** the statistical distribution of metastasis numbers. For **c**, **d**, **g** and **h**, the date were expressed as mean ± SD in three independent experiments. *P < 0.05, **P < 0.01

To further confirm our in vitro findings, we performed an in vivo tumourigenesis experiment in nude mice (Fig. 5a). Tumour volumes, growth rates and weights were significantly decreased in tumours created by HOXD-AS1-overexpressing SW620 cells compared to those created by control transfected SW620 cells (Fig. 5b-d). The HOXD-AS1-overexpressing tumours also raised H3K27me3 protein level and reduced HOXD3 expression and Ki-67 index in xenografts by IHC (Fig. 5e). These results suggest that HOXD-AS1 exerts a significant inhibitory effect on tumourigenesis in vivo. In addition, we performed intrasplenic injections to establish a liver metastasis model in nude mice and found that the HOXD-AS1 overexpression group exhibited a decrease in the number of definite liver colonization sites compared with the control group (Fig. 5f, g and h). Consistent with the in vitro results, these data also indicated an important inhibitory role for HOXD-AS1 in CRC growth and metastasis in vivo. In contrast, the overexpression of HOXD3 attenuated the proliferation and metastatic potential of the HOXD-AS1-overexpressing SW620 cells to levels similar to those of the control cells (Fig. 5). These results suggest that HOXD3 is specifically required for HOXD-AS1 to affect SW620 cell behaviours.

### HOXD3 promotes integrin β3 transcription and activates the MAPK/AKT signalling pathways

**Fig. 6:**
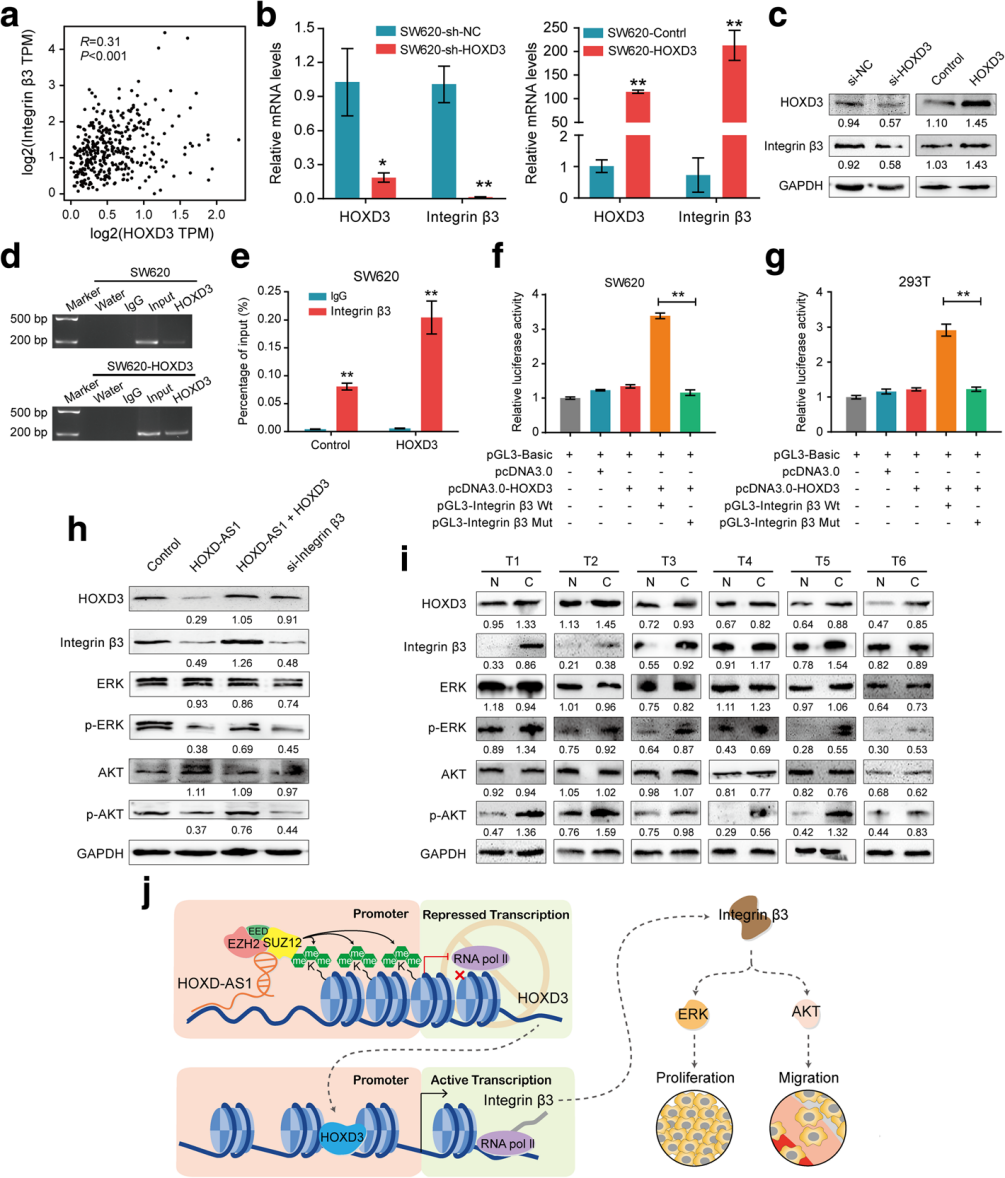
HOXD3 mediates Integrin β3 expression by activating Integrin β3 transcription. **a** A positive correlation between HOXD3 and Integrin β3 expression was found in the TCGA cohort. **b**–**c** The expression of both integrin β3 **b** mRNA and **c** protein was significantly up-regulated after HOXD3 ectopic overexpression in SW620 cells, while the expression of integrin β3 was reduced when HOXD3 knockdown was mediated by a siRNA. **d** ChIP assays were performed in HOXD-AS1 overexpressed(SW620-HOXD-AS1)and control cells using anti-HOXD3- antibodies or IgG antibodies, respectively. **e** The enrichments of HOXD3 of the region upstream of the Integrin β3 gene were examined by real-time PCR. **f**-**g** Dual-luciferase report showed that HOXD3 activated Integrin β3 transcription both in **f** SW620 and **g** 293 T cells. **h** Western blot was performed to detect HOXD3, Integrin β3, ERK, p-ERK, AKT and p-AKT expression in SW620-HOXD-AS1, SW620-HOXD-AS1 + HOXD3, SW620-si-Integrin β3 and control cells. **i** Western blot was performed to detect HOXD3, Integrin β3, ERK, p-ERK, AKT and p-AKT expression in paired CRC tissues and adjacent non-cancerous tissues. **j** A proposed model for illustrating the function and mechanism of HOXD-AS1 in CRC growth and metastasis**.** For b, e, f and g, data were expressed as means ± SD in three independent experiments. *P < 0.05, **P < 0.01

Previous studies have shown that the overexpression of HOXD3 contributes to the enhancement of the expression of Integrin β3 in A549 cells [29, 30], however, the underlying mechanism has not been clearly defined. Interestingly, as shown in Fig. 6a, a positive correlation between HOXD3 and Integrin β3 expression was found in the TCGA cohort (*n* = 367, *R* = 0.31, *P* < 0.001). We also found that the expression of both Integrin β3 mRNA and protein was significantly up-regulated after HOXD3 ectopic overexpression in SW620 cells, while the expression of Integrin β3 was reduced when HOXD3 knockdown was mediated by a siRNA (Fig. 6b and c). These results indicate that HOXD3 could regulate Integrin β3 expression at the transcriptional level.

Next, we predicted that there is one site for HOXD3 binding in the Integrin β3 promoter (Additional file 2: Table S5) using the JASPAR online database [31]. Consistent with this prediction, ChIP analysis revealed significant occupancy by HOXD3 of the region upstream of the Integrin β3 gene (Fig. 6d and e). Moreover, a dual-luciferase report showed that HOXD3 activated Integrin β3 transcription both in SW620 and 293 T cells (Fig. 6f and g), suggesting that HOXD3, as a transcription factor, is involved in the regulation of Integrin β3 expression.

Integrin β3 has been reported to induce the MAPK/ERK signalling pathway to promote proliferation and activate the AKT signalling pathway to promote metastasis in cancer [32, 33], thus, we sought to examine this effect mediated by HOXD-AS1 in CRC. The overexpression of HOXD-AS1 significantly reduced the levels of p-AKT and p-ERK. However, this reduction was completely attenuated to levels similar to those of the control cells when HOXD3 was ectopically overexpressed. Meanwhile, Integrin β3 depletion also rescued this inhibited effect in the HOXD3- overexpressing SW620 cells (Fig. 6h). Moreover, in human CRC tissues, the up-regulation of HOXD3 could increase the levels of Integrin β3, p-EAR and p-AKT proteins, compared to adjacent nontumor tissues (Fig. 6i). Importantly, we also observed that changes in HOXD3 abundance specifically affected the activity of MAPK/ERK signalling through Integrin β3 in the xenografts tissues in vivo (Additional file 8: Figure S6).

To test the contribution of MAPK/ERK and AKT signalling pathways on the important role of HOXD-AS1, we inactivated the MAPK/ERK and AKT signalling in HOXD-AS1 depletion CRC cells with inhibitor of ERK (SCH772984) or AKT (LY294002), respectively (Additional file 9: Figure S7a). The down-regulation of HOXD-AS1 significantly promoted cell proliferation, cell-cycle progression and migration in SW480 and DLD-1 cells. However, this potential was completely abolished to the levels similar to the control cells when using SCH772984. These results suggest that the activation of MAPK/ERK signalling is required for HOXD-AS1 to affect CRC cell behaviours (Additional file 9: Figure S7b-f). Similar results were obtained using LY294002 AKT inhibitor (Additional file 9: Figure S7b-f). Collectively, these results suggest that HOXD-AS1 regulates CRC progression through the MAPK/AKT signalling pathways, which is mediated by the HOXD3-Integrin β3 axis (Fig. 6j).

## Discussion

The key finding of this study is that the lncRNA HOXD-AS1 was expressed in the nucleolus of CRC cells and was commonly downregulated in most CRC specimens and CRC cell lines. A lower level of HOXD-AS1 expression was associated with an advanced stage and poor prognosis in CRC patients. Furthermore, HOXD-AS1 depletion contributed to cell proliferation, invasion and metastasis. Interestingly, the downregulation of HOXD-AS1 expression decreased PRC2 recruitment, which resulted in the reduction in H3K27me3 levels and HOXD3 transcriptional activation. In turn, HOXD3, as a transcriptional activator, promoted Integrin β3 transcription, which subsequently activated the MAPK/AKT signalling pathways. Thus, our results reveal a novel mechanism of the HOXD-AS1/HOXD3/Integrin β3 regulatory axis involved in epigenetic and transcriptional regulation.

Previous studies have shown that HOXD-AS1 is expressed in multiple tissues with the highest levels of expression in the kidneys, colon and testes and the lowest in the liver, heart, pancreas and stomach [8]. However, the expression patterns of HOXD-AS1 in various kinds of cancers remain controversial. In neuroblastoma cells [8], HOXD-AS1 is strongly induced by retinoic acid (RA) treatment, a differentiation agent that is the first choice drug for NB therapy [34]. HOXD-AS1 overexpression is noted in ovarian cancer [10], glioma [11], bladder cancer [13], and NSCLC [14]. Although the HOXD-AS1 level is significantly higher in prostate cancer patients with a Gleason score of 7(4 + 3)-10 compared with those with a Gleason score of 6–7(3 + 4), the expression level of HOXD-AS1 did not change significantly between benign tissue and cancer tissue [35]. In contrast, HOXD-AS1 was shown to be one of the most drastically downregulated genes in hepatocellular carcinoma [36]. Li et al. demonstrated that HOXD-AS1 expression was upregulated in CRC by testing cancer tissue blocks using real-time PCR [16]. However, in our study based on cell lines and a larger number of clinical samples using real-time PCR and ISH, a significantly higher incidence rate of HOXD-AS1 expression downregulation was found in CRC. Consistent with our results, the downregulation of HOXD-AS1 expression has been found in seven unique public databases. In addition, Sabates-Bellver et al. found that HOXD-AS1 expression is strongly suppressed in colorectal adenoma, discriminating these precancerous lesions from the surrounding normal mucosa tissue [19]. We also determined that the low expression levels of HOXD-AS1 were significantly associated with poor differentiation, advanced staging and cancer-related death in CRC patients, which consisted with the HOXD-AS1 expression up-regulation induced by RA, accompanying with growth arrest and differentiation in NB cells [34]. These findings together support the hypothesis that the downregulation of HOXD-AS1 expression might be common in CRC.

HOXD-AS1 has been shown to promote cell proliferation, migration and invasion in some cancers [10–14]. In the present study, the downregulation of HOXD-AS1 expression indicated a potential suppressor function in CRC. Indeed, using gain- and loss-of-function experiments, our data clearly indicated that HOXD-AS1 deficiency not only promotes cell proliferation and migration in in vitro cultured CRC cells but also promotes in vivo tumour growth and metastasis in xenograft tumour models. While this manuscript was in preparation, Li et al. reported that HOXD-AS1 overexpression promotes CRC cell growth and metastasis [16]. A possible explanation for the discrepancy is that a difference in HOXD-AS1 subcellular localization resulted in the different functions in CRC. Our work presented here and the work by Gu et al. [35] showed that HOXD-AS1 is located mainly in the nucleus. Although they did not perform RNA fraction assays, Li et al. [16] found that HOXD-AS1 and miR-217 were found in the same RNA-induced silencing complex, which indicated that HOXD-AS1 was distributed mainly in the cytoplasm of CRC cells. Additional experimentation will be needed to clarify the issue of HOXD-AS1 nucleus-cytoplasmic shuttling and the possible mechanisms.

The subcellular localization of a lncRNA is closely associated with its biological function. Nuclear-enriched lncRNAs are involved in key cellular processes, including epigenetic regulation, chromosomal interactions, and transcriptional regulation [37]. LncRNAs enriched in the cytoplasm typically participate in posttranscriptional regulation by interacting with microRNAs or mRNAs [38]. Previous studies found that HOXD-AS1 acted as a ceRNA that sponges up miRNAs to regulate gene expression [10–15]. Here, HOXD-AS1 was determined to be enriched in the nucleus of CRC cells based on ISH, FISH and RNA fraction assays, which is consistent with the results reported in prostate cancer [35]. We also demonstrated that HOXD-AS1 could bind with the SUZ12 and EZH2 proteins, two members of the PRC2 complex. Polycomb group (PcG) genes are key epigenetic regulators in multicellular organisms, as they maintain the transcriptional repression of genes throughout development and tumourigenesis [39]. Because none of the core components of PRC2 possess a DNA-binding domain, it is believed that chromatin targeting must be specified elsewhere by interactions with DNA-binding factors [40]. LncRNAs appear well suited to exchanging information between chromatin-modifying complexes and the genomic sequence [41]. Approximately 20% of lncRNAs could interact with the PRC2 complex [42], such as HOXAIR [43, 44]. Notably, changes in HOXD-AS1 abundance that occur through ectopic overexpression or siRNA-mediated knockdown have specifically affected the expression of HOXD3, a sense-cognate gene for HOXD-AS1, through PRC2 recruitment, along with changes in the level of repressive H3K27me3. Most importantly, the change in HOXD-AS1 expression did not affect the expression of HOXD1, another sense-cognate gene for HOXD-AS1. These data suggest a specific epigenetic regulation mechanism at the histone level that is driven by HOXD-AS1 in CRC.

The genes of the HOX family are conserved transcription factors that determine cellular identity during development. Numerous studies have shown that dysregulated HOX expression plays a regulatory role in malignancy [45]. HOXD3 is the third paralog of the HOXD family and has been shown to play a pivotal role in cancer cell invasion, metastasis, and angiogenesis [26, 28, 29, 46]. In the present study, we demonstrated that HOXD-AS1 could negatively control the expression levels of HOXD3 mRNA and protein. Indeed, in contrast to the HOXD-AS1 expression pattern in CRC, endogenous HOXD3 expression was considerably higher in most CRC tissues and all CRC cell lines. The high expression of HOXD3 protein was positively associated with clinicopathologic features, including lymph node metastasis status, distant metastasis status, and poor outcome in CRC patients. Furthermore, we applied gain- and loss-of-function methods to reveal the involvement of HOXD3 in CRC. To our knowledge, we determined for the first time that HOXD3 facilitates CRC growth and metastasis in vitro and in vivo*.* Our results, along with those of previous studies, indicate that HOXD3 promotes carcinogenesis and progression. Most importantly, the inhibitory effects exerted by HOXD-AS1 on cell growth and metastasis are rescued by HOXD3 overexpression, especially in vivo. Thus, our results revealed that the inhibitory effects of HOXD-AS1 on CRC progression require HOXD3.

HOXD3 plays a role in enhancing invasion and metastasis by coordinating the expression of metastasis-associated factors. Integrin β3, a well-known member of the Integrin family, has been shown to be extensively involved in cell proliferation and metastasis in various cancers [47–50]. Moriuchi group found that overexpression of the HOXD3 gene enhanced Integrin β3 expression in both human erythroleukaemia HEL cells and lung carcinoma A549 cells [29, 30]. In breast cancer, the expression of HOXD3 was closely associated with Integrin β3 expression [26]. However, the mechanism involved in the regulation of Integrin β3 by HOXD3 has not been clearly defined. Given that HOXD3 is a transcription factor, we predicted the HOXD3 binding sites within the Integrin β3 promoter. Furthermore, we found that HOXD3 could indeed occupy the upstream region of the Integrin β3 gene and activate Integrin β3 transcription. Thus, the present results demonstrated that HOXD3, as a transcription factor, is involved in the regulation of Integrin β3 expression. Moreover, we further revealed that HOXD3 overexpression activated MAPK/ERK and AKT signalling by enhancing Integrin β3 expression, thereby enhancing CRC growth and metastasis, which clarified the mechanism by which the downregulation of HOXD-AS1 expression promoted CRC progression.

## Conclusions

Taken together, we established a previously unknown function for nuclear HOXD-AS1 in CRC. The effects of HOXD-AS1 on cell proliferation, migration and invasion suggest that HOXD-AS1 inhibits the tumourigenesis and progression of CRC. We also provide evidence that the transcription factor HOXD3 may represent a downstream effector of HOXD-AS1. HOXD-AS1 participates in CRC progression by repressing HOXD3-dependent Integrin β3 transcription and thereby inactivating the MAPK/AKT pathways. The development of HOXD-AS1-based therapeutic strategies may provide a novel therapeutic approach for CRC treatment.

## Additional files

Additional file 1: Supplementary materials and methods. (DOCX 23 kb)

Additional file 2: **Table S1.** Summary of overall survival analysis by univariate and multivariate COX regression analysis. **Table S2.** Mass spectrometry analysis of the proteins pulled down by HOXD-AS1. **Table S3.** Summary of overall survival analysis by univariate and multivariate COX regression analysis. **Table S4.** Bioinformatics predict the binding sites of Integrin β3 with HOXD3. **Table S5.** Oligonucleotide sequences in this study. (XLSX 27 kb)

Additional file 3: **Figure S1.** Nuclear HOXD-AS1 expression is reduced in CRC. The analysis of HOXD-AS1 expression in CRC compared with normal tissues in CRC microarray profile (GES32323, Wilcoxon matched-pairs signed rank test; GSE41328, Paired t test; GSE23878, t test; GSE9348, t test). (TIF 477 kb)

Additional file 4: **Figure S2**. HOXD-AS1 has no obvious regulatory effect on HOXD1 expression, a sense-cognate gene for HOXD-AS1. (a) Analysis of genes adjacent to HOXD-AS1 in the UCSC database, and found that HOXD-AS1 is located between HOXD1 and HOXD3. (b) Real-time PCR was used to detect the expression of HOXD1 in HOXD-AS1-overexpressed or -depleted CRC cells, respectively. For b, data were expressed as means ± SD in three independent experiments. n.s: P > 0.05. (TIF 2214 kb)

Additional file 5: **Figure S3.** HOXD3 possesses oncogenic functions in CRC. (a) Real-time PCR analysis of HOXD3 expression in CRC cell lines and normal cell line (FHC). HOXD3 level was normalized to GAPDH expression. (b) HOXD3-overexpressing HCT116 and DLD-1 cell lines were established by the transfection of pcDNA3.0-HOXD3. Real-time PCR (upper) and Western blot (down) were performed to detect the expression of HOXD3. (c) CCK-8 assays were performed to determine the proliferation of HOXD3-overexpressed CRC cells. (d) Colony-forming assays were performed to determine the effects of HOXD3 overexpression on the growth of CRC cells. The diameter > 50 cells was scored. (e) Cell cycle progression was analyzed by flow cytometry. (f) The migration potencies of CRC cells with the indicated treatments were detected by using wound healing assay. (g) Invasion assays were used to determine the effects of HOXD3 overexpression on the invasion ability of CRC cells. For a-g, data were expressed as means ± SD in three independent experiments. *P < 0.05, **P < 0.01, ***P < 0.001. (TIF 5824 kb)

Additional file 6: **Figure S4.** HOXD-AS1 regulates HOXD3 expression through cooperating with PRC2 complex. (a) RIP assays were performed in SW620 cells using anti-SUZ12- antibodies, anti-EZH2- antibodies or nonspecific IgG antibodies respectively. Real-time PCR was performed to determine amount of RNA associated with SUZ12, EZH2 or IgG compared with the input control. (b) ChIP assays were performed in HOXD-AS1 overexpressed(SW620-HOXD-AS1)and control cells using anti-EZH2, anti-SUZ12, anti-H3K27me3 or IgG antibodies respectively. The ChIP products were amplified by real-time PCR. (TIF 3699 kb)

Additional file 7: **Figure S5.** HOXD3 is required for the HOXD-AS1-mediated progress of CRC in vitro. (a) Real-time PCR analysis of HOXD3 expression in SW620-HOXD-AS1, SW620-HOXD-AS1 + HOXD3 and control cells. HOXD3 level was normalized to GAPDH expression. (b) CCK-8 assay, (c) colony formation assay and (d) cell cycle progression assay were performed to determine the cell proliferative ability. (e) Wound healing assay and (f) Transwell assay were used to examine the migratory and invasive abilities of CRC cells. For a-f, the date were expressed as mean ± SD in three independent experiments. *P < 0.05, **P < 0.01, ***P < 0.001. (TIF 5471 kb)

Additional file 8: **Figure S6.** Examine the expression of HOXD3 and Integrin β3/MAPK/AKT signaling in xenografts by IHC assays. (TIF 9353 kb)

Additional file 9: **Figure S7.** HOXD-AS1 regulates CRC progression through the MAPK/AKT signaling pathways. (a) Detected AKT, p-AKT, ERK, p-ERK protein level in SW480 and DLD-1 cells after being treated with inhibitor of ERK (SCH772984) or AKT (LY294002), respectively. CCK-8 assay (b) colony formation assay (c) and cell cycle progression assay (d) were performed to determine the cell proliferative ability of CRC cells. (e) Wound healing assay and (f) Transwell assay were used to examine the migratory and invasive abilities of CRC cells. For b-f, the date were expressed as mean ± SD in three independent experiments. *P < 0.05, **P < 0.01, ***P < 0.001. (TIF 9210 kb)

## Abbreviations

ceRNA: Competing endogenous RNA
ChIP: Chromatin immunoprecipitation
CRC: Colorectal carcinoma
EZH2: Ehancer of Zeste homologue 2
GEO: Gene expression omnibus
H3K27me3: Histone 3 lysine 27
HOXD-AS1: HOXD cluster antisense RNA1
IHC: Immunohistochemistry
ISH: in situ hybridization
lncRNAs: long noncoding RNAs
NSCLC: Non-small cell lung cancer
PcG: Polycomb group
PRC2: Polycomb repressive complex
RIP: RNA immunoprecipitation
SUZ12: Zeste 12 homologue
TCGA: the cancer genome atlas
Tiam1: T lymphoma invasion and metastasis gene
