# Supplementary material for: Nuclear lncRNA HOXD-AS1 suppresses colorectal carcinoma growth and metastasis via inhibiting HOXD3-induced integrin β3 transcriptional activating and MAPK/AKT signalling
Source: Mol Cancer. 2019 Mar 1;18:31. doi: 10.1186/s12943-019-0955-9 (PMC6397497; doi:10.1186/s12943-019-0955-9)
Supplement: Supplementary file 1 — Supplementary materials and methods. (DOCX 23 kb) [file 12943_2019_955_MOESM1_ESM.docx]

**Supplementary materials and methods**

**Clinical specimens and cell culture**

All CRC specimens and matched adjacent non-tumour tissues were obtained with informed consent from the patients who were diagnosed with CRC and subsequently carried out surgery at Nanfang Hospital, Southern Medical University (Guangzhou, China). Freshly frozen tumor samples from 35 CRC patients were selected for real-time PCR. Formalin-fixed tumor tissue samples, 164 CRC tumor tissues were used for immunohistochemistry (IHC) and for *in situ* hybridization (ISH). Complete follow-up, ranging from 1-117 months, was achieved for all patients, and the median survival time was 56 months. No patient received any pre-operative chemotherapy and radiotherapy.

The human CRC cell lines (SW620, SW480, DLD-1, HCT116, and LoVo), normal colon epithelial cell line FHC, and the human embryonic kidney 293T cells were obtained from American Type Culture Collection (ATCC, Manassas, VA, USA). M5, a subclone with enhanced metastasis ability, was derived from SW480 through *in vivo* selection in our laboratory[61]. All of the CRC cell lines were cultured in PRMI 1640 medium (Gibco, Gaithersburg, MD, USA) with 10% fetal bovine serum (Hyclone, Logan, USA) at 37°C in a humidified chamber containing 5% CO_2_. HEK-293T was cultured in Dulbecco’s modified Eagle’s medium (DMEM; Gibco, Gaithersburg, MD, USA) supplemented with 10% FBS.

**RNA isolation, reverse transcription, and real-time PCR**

Total RNA samples from CRC tissue specimens and cell lines were respectively extracted using TRIzol Reagent (Takara, Dalian, China). Cytoplasmic and nuclear RNA were isolated and purified using the Protein and RNA Isolation System Kit (Ambion by life Technologies, Carlsbad, CA, USA) following the manufacturer’s instructions. The cDNA was synthesized using the PrimeScript RT Reagent Kit (TaKaRa, Dalian, China) according to the manufacturer’s protocol. Real-time PCR was performed with SYBR^®^ Premix Ex Taq^TM^ II (TaKaRa, Dalian, China). GAPDH was used as the endogenous control. The relative expression of RNAs was normalized to internal controls and calculated using the comparative Ct method (2^-ΔΔCT^). The sequences used for the gene special primer were listed in the Supplementary Table 5.

***In situ* hybridization and evaluation of HOXD-AS1 staining.**

HOXD-AS1 expression levels were measured in the CRC tissues and non-tumour tissues by ISH according to the manufacturer’s instructions. Briefly, after deparaffinized, rehydrated, and digested with proteinase K, the samples were hybridized with the 5′-digoxin-labeled probes of HOXD-AS1(HongTu, Guangzhou, China) at 42 °C overnight and subsequently incubated for 30min at 30°C with anti-Digoxigenin-AP-Fab fragments (Roche Diagnostics, Indianapolis, IN, USA). The sequence of HOXD-AS1 probes are listed in Supplementary Table 5.

All slides were reviewed and scored independently by two pathologists without knowledge of HOXD-AS1 and clinical outcome. The staining scores were determined using a relatively simple, reproducible scoring method based on both the intensity (0, no staining; 1, weak staining; 2, medium staining; 3, strong staining) and proportion of HOXD-AS1-positive cells (0, 0%; 1, 1–25%; 2, 26–50%; 3, 51–75%; 4, 76–100%). The final staining score of HOXD-AS1 was calculated by multiplying the staining intensity score by staining extent score. Some slides with inhomogeneous expression would score extra points. The final staining score of ≥3 was considered to be high.

**RNA fluorescence *in situ* hybridization**

The HOXD-AS1 probes were designed and produced by HongTu (Guangzhou, China). After cultured in confocal plates overnight, cells were fixed for 30 min with 4% paraformaldehyde and treated for 10 min using 0.5% Triton X-100. After blocked with blocking buffer for 30 min, the plates were incubated with the special HOXD-AS1 probes and then with anti-Digoxigenin-Fluorescein-Fab fragments (Roche Diagnostics, Indianapolis, IN, USA). The slides were mounted with Prolong Gold Antifade Reagent with 40, 6-diamidino-2-phenySlindole (DAPI, Invitrogen, Carlsbad, CA) for detection. The localization of HOXD-AS1 was observed under an Olympus FluoView™ FV1000 confocal microscope (Olympus, Hamburg, Germany).

**Western blot**

Protein lysates from cells was prepared in 1 × sodium dodecyl sulfate buffer, separated by 10% SDS-PAGE, and transferred to a nitrocellulose membrane (Sigma, Shanghai, China). The membranes were blocked with 5% non-fat milk and subsequently incubated with the appropriate antibodies. The antibodies used were as follow: HOXD3 (rabbit polyclonal, ab22840, abcam, Cambridge, UK, dilution: 1:1000), ITGB3 (rabbit monoclonal, ab179473, abcam, Cambridge, UK, dilution: 1:1000), SUZ12 (rabbit polyclonal, ab12073, abcam, Cambridge, UK, dilution: 1:1000), EZH2 (rabbit monoclonal, #5242, CST, Danvers, MA, USA, dilution: 1:1000), H3K27me3 (rabbit monoclonal, #8352, CST, Danvers, MA, USA, dilution: 1:1000), AKT (rabbit monoclonal, #4691, CST, Danvers, MA, USA, dilution: 1:1000), Phospho-AKT (rabbit monoclonal, #4060, CST, Danvers, MA, USA, dilution: 1:1000), ERK (rabbit monoclonal, #9107, CST, Danvers, MA, USA, dilution: 1:1000), Phospho-ERK (rabbit monoclonal, #4370, CST, Danvers, MA, USA, dilution: 1:1000). The protein levels were normalized with that of GAPDH (mouse monoclonal, 60004-1-Ig, Proteintech Group Inc., Wuhan, China, dilution: 1:1000) or histone H3 (rabbit monoclonal, 17168-1-AP, Proteintech Group Inc., Wuhan, China, dilution: 1:1000) The proteins were visualized by chromogenic substrate and quantified by densitometry.

**Immunohistochemistry**

IHC was performed following the manufacturer’s protocol. Briefly, after deparffinized and rehydrated, the samples were treated with 3% H_2_O_2_ for 10 minutes to eliminate intrinsic peroxidase activity, and then incubated overnight at 4 °C with primary antibodies. After washing and incubated with the biotinylated secondary antibodies (dako, Denmark), the sections were visualized with 3, 3’-iaminobenzidine DAB and counterstained with hematoxylin, and then dehydrated and mounted. The antibodies used were as follow: rabbit antibody to HOXD3 (ab22840, abcam, Cambridge, UK, dilution: 1:250), rabbit antibody to ITGB3 (ab179473, abcam, Cambridge, UK, dilution: 1:300), and mouse antibody to Ki-67 (27309-1-AP, Proteintech, Wuhan, China, dilution 1:100).

To assess the expression of HOXD3, the IHC sides for HOXD3 were reviewed and scored independently by two pathologists. The score principle in IHC was the same as ISH. The final staining score of ≥3 was considered to be high.

**Construction of cell lines with stably downregulated HOXD-AS1 and HOXD3**

There shRNA sequence (Supplementary Table 5) specially targeting HOXD-AS1 or HOXD3 were designed and synthesized, and clone into a pGU6/GFP/Neo-shRNA vector (GenePharma, Shanghai, China). The most effective shRNA sequence in achieving knockdown of HOXD-AS1 or HOXD3 expression was selected to be constructed into lentiviruses by GenePharma (Suzhou, China). A scrambled shRNA oligo, namely, the pGU6/GFP/control, was used as a control. For lentiviral transfection, 5x10^4^ cells were mixed with 1ml medium, 5 µg/ml polybrene, and 2ul LV- HOXD-AS1(9 ×10^8^ TU/ml), or 5µl HOXD3 (8×10^8^ TU/ml), or 5ul LV-NC(5 ×10^8^ TU/ml). After 24 hours, transfected cells were selected with 2 µg/ml puromycin (Sigma-Aldrich), and then maintained in growth medium with1 µg/ml puromycin.

**Cell Proliferation, flow cytometry cell cycle assay** **and colony formation assays**

For cell proliferation assay, cells were seeded into 96-well plates with 1×10^3^ cells per well. After 24 hours of culture, the cells proliferation ability was evaluated using the CCK-8 (Dojindo, Rockville, MD, USA) according to the manufacturer’s protocol.

For cell flow cytometry cell cycle assay, cells were plated in 6-well plates at 5×10^5^ per well. The cell-cycle distribution was analyzed by propidium iodide (Sigma-Aldrich) staining and flow cytometry. All experiments were performed in triplicates.

For colony formation assay, cells were plated in 6-wall plates at 5×10^2^ cells per well and incubated with 10%FBS for 2 weeks. After 2 weeks, clones were fixed with methanol and stained with Giemsa, and the number of colonies was counted under a microscope.

**Wound-healing and invasion assays**

For wound healing assay, acellular area was created by a 10 μl pipette tube, and the spread extent of wound closure was observed after 0 and 48 hours, respectively. The migratory ability was quantified by counting the total number of cells that migrated toward the original wound field.

For the invasion assay, a final concentration of 1×10^5^ cells/ml were seeded into the upper chambers (coated in Matrigel) in serum-free medium. The lower chamber of the Transwell was filled with culture media containing 10% FBS as a chemoattractant. After the chambers were incubated at 37 °C for 48 hours, successfully translocated cells were fixed with 10% formalin. Then, they were stained with 0.1% crystal violet for 30 min and counted under a light microscope.

***In vivo* tumorigenic and metastasis assays**

Balb/C-nu/nu nude mice (4 weeks old) were purchased from the Laboratory Animal Center of Southern Medical University. The animals were fed with an autoclaved laboratory rodent diet.

For *in vivo* tumorigenicity, a total 5×10^6^ SW620 cells stably with HOXD-AS1 and HOXD3 overexpression, or SW620 transfected with HOXD-AS1 vector, or mock cells, were subcutaneously injected into the right and left bilateral upper limbs of mice respectively. Tumors were allowed to grow for 24 days before the mice were killed by cervical dislocation, and tumors were dissected and weighted. Tumor volume was recorded every three days, and calculated according to the formula 0.5 × length × wildth^2^.

The *in vivo* metastasis assays were conducted using intrasplenic injection with 5×10^6^ cells. After 6 weeks, the mice were killed by cervical dislocation. The liver was removed by dissection away from adjacent organs, and fixed using 10% neutral-buffered formalin. Subsequently, the consecutive tissue sections were obtained and stained with hematoxylin-eosin (H&E) to observe the metastatic nodules of the livers under the microscope. The colonization number was counted.

**Inhibitors**

Inhibitors including AKT inhibitor LY294002 (Cell Signal Technology, Danvers, MA), ERK inhibitor SCH772984 (Selleck, Shanghai, China) were added to cultured CRC cells for 72h at a concentration of 10 μM. Cells treated with inhibitors were prepared for the following western blotting and function experiments.
