# Supplementary figures and images for: Nuclear lncRNA HOXD-AS1 suppresses colorectal carcinoma growth and metastasis via inhibiting HOXD3-induced integrin β3 transcriptional activating and MAPK/AKT signalling
Source: Mol Cancer. 2019 Mar 1;18:31. doi: 10.1186/s12943-019-0955-9 (PMC6397497; doi:10.1186/s12943-019-0955-9)

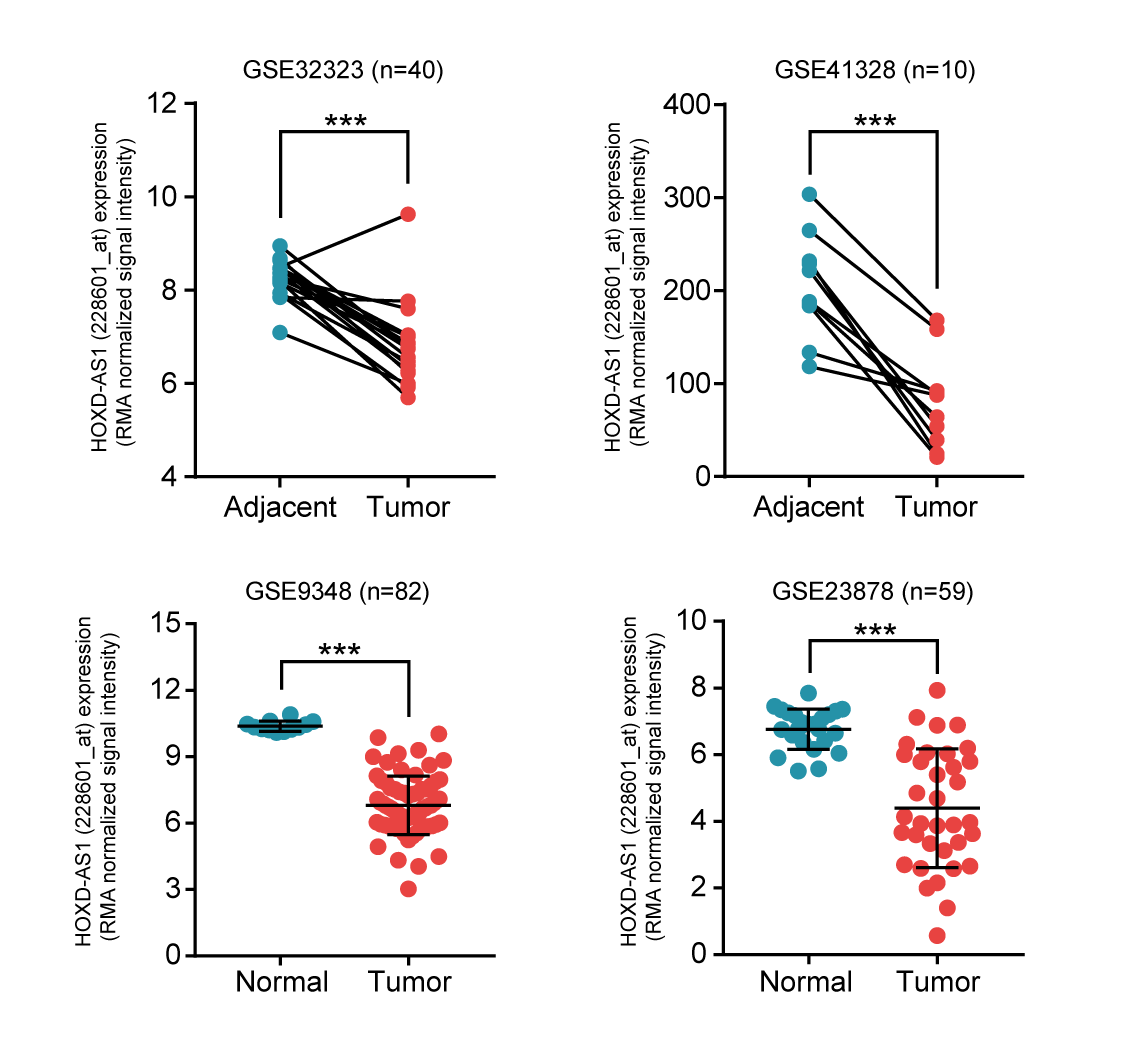

Supplement: Supplementary file 3 — Figure S1. Nuclear HOXD-AS1 expression is reduced in CRC. The analysis of HOXD-AS1 expression in CRC compared with normal tissues in CRC microarray profile (GES32323, Wilcoxon matched-pairs signed rank test; GSE41328, Paired t test; GSE23878, t test; GSE9348, t test). (TIF 477 kb) [file 12943_2019_955_MOESM3_ESM.tif]

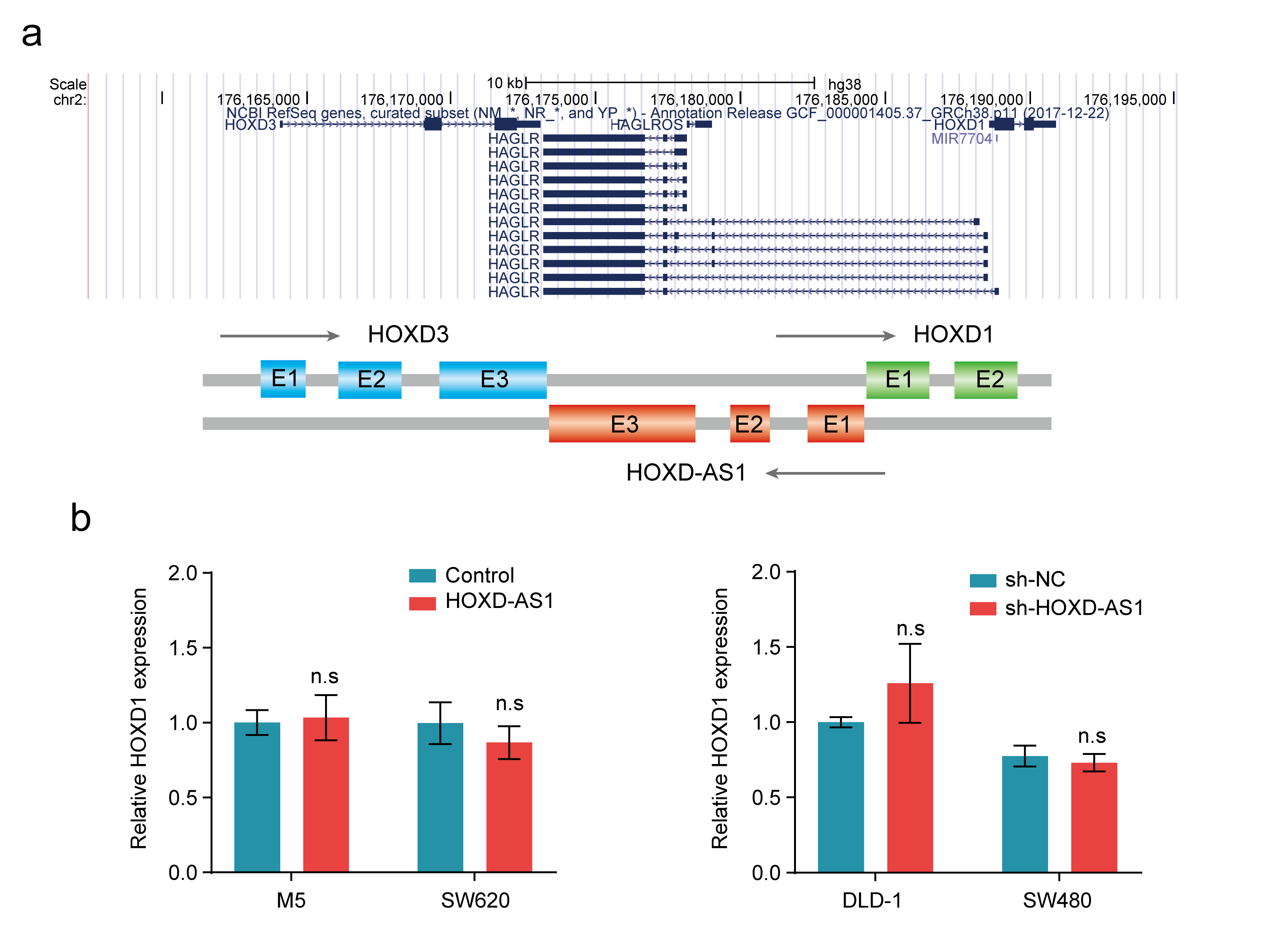

Supplement: Supplementary file 4 — Figure S2. HOXD-AS1 has no obvious regulatory effect on HOXD1 expression, a sense-cognate gene for HOXD-AS1. (a) Analysis of genes adjacent to HOXD-AS1 in the UCSC database, and found that HOXD-AS1 is located between HOXD1 and HOXD3. (b) Real-time PCR was used to detect the expression of HOXD1 in HOXD-AS1-overexpressed or -depleted CRC cells, respectively. For b, data were expressed as means ± SD in three independent experiments. n.s: P > 0.05. (TIF 2214 kb) [file 12943_2019_955_MOESM4_ESM.tif]

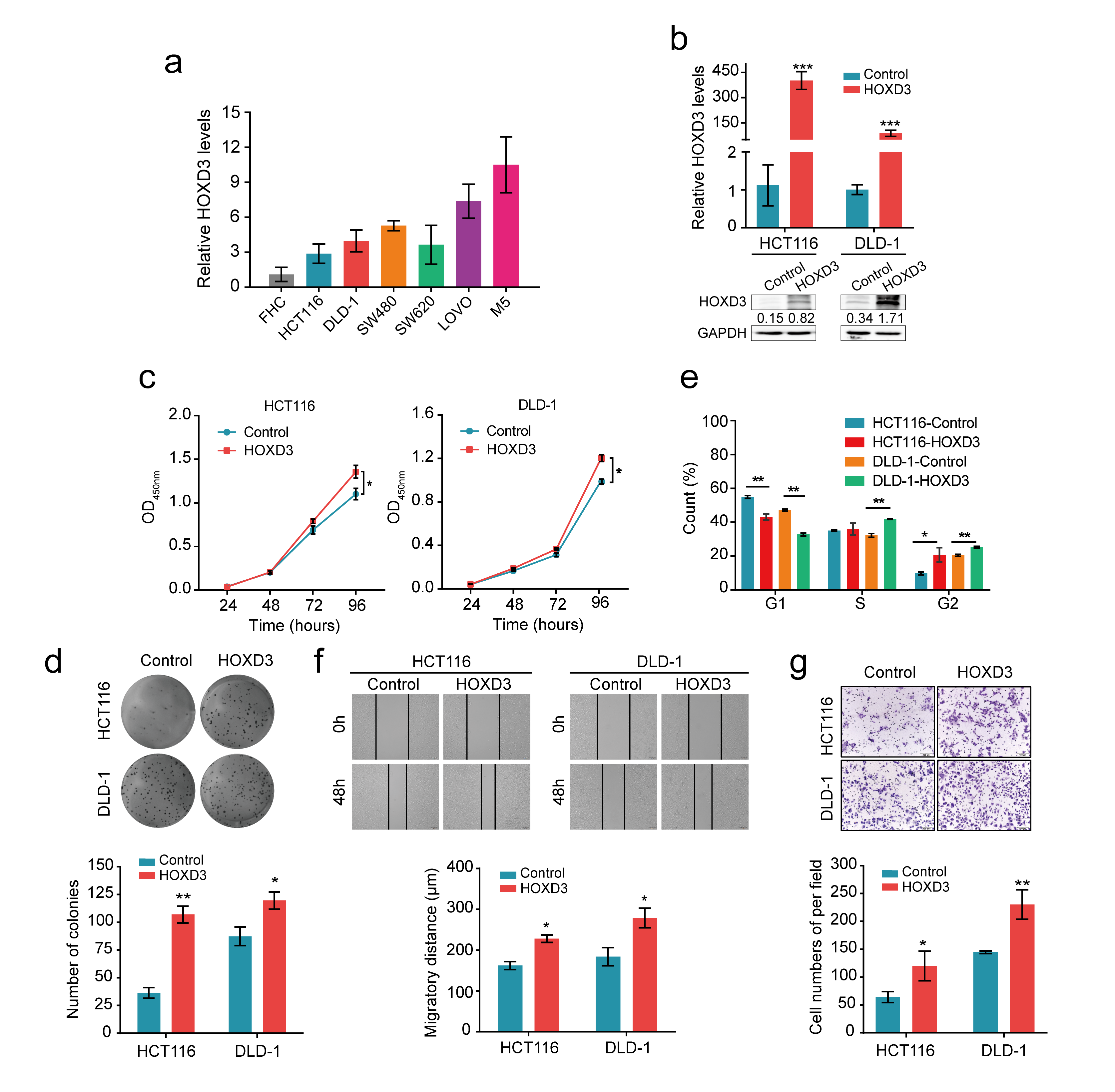

Supplement: Supplementary file 5 — Figure S3. HOXD3 possesses oncogenic functions in CRC. (a) Real-time PCR analysis of HOXD3 expression in CRC cell lines and normal cell line (FHC). HOXD3 level was normalized to GAPDH expression. (b) HOXD3-overexpressing HCT116 and DLD-1 cell lines were established by the transfection of pcDNA3.0-HOXD3. Real-time PCR (upper) and Western blot (down) were performed to detect the expression of HOXD3. (c) CCK-8 assays were performed to determine the proliferation of HOXD3-overexpressed CRC cells. (d) Colony-forming assays were performed to determine the effects of HOXD3 overexpression on the growth of CRC cells. The diameter > 50 cells was scored. (e) Cell cycle progression was analyzed by flow cytometry. (f) The migration potencies of CRC cells with the indicated treatments were detected by using wound healing assay. (g) Invasion assays were used to determine the effects of HOXD3 overexpression on the invasion ability of CRC cells. For a-g, data were expressed as means ± SD in three independent experiments. *P < 0.05, **P < 0.01, ***P < 0.001. (TIF 5824 kb) [file 12943_2019_955_MOESM5_ESM.tif]

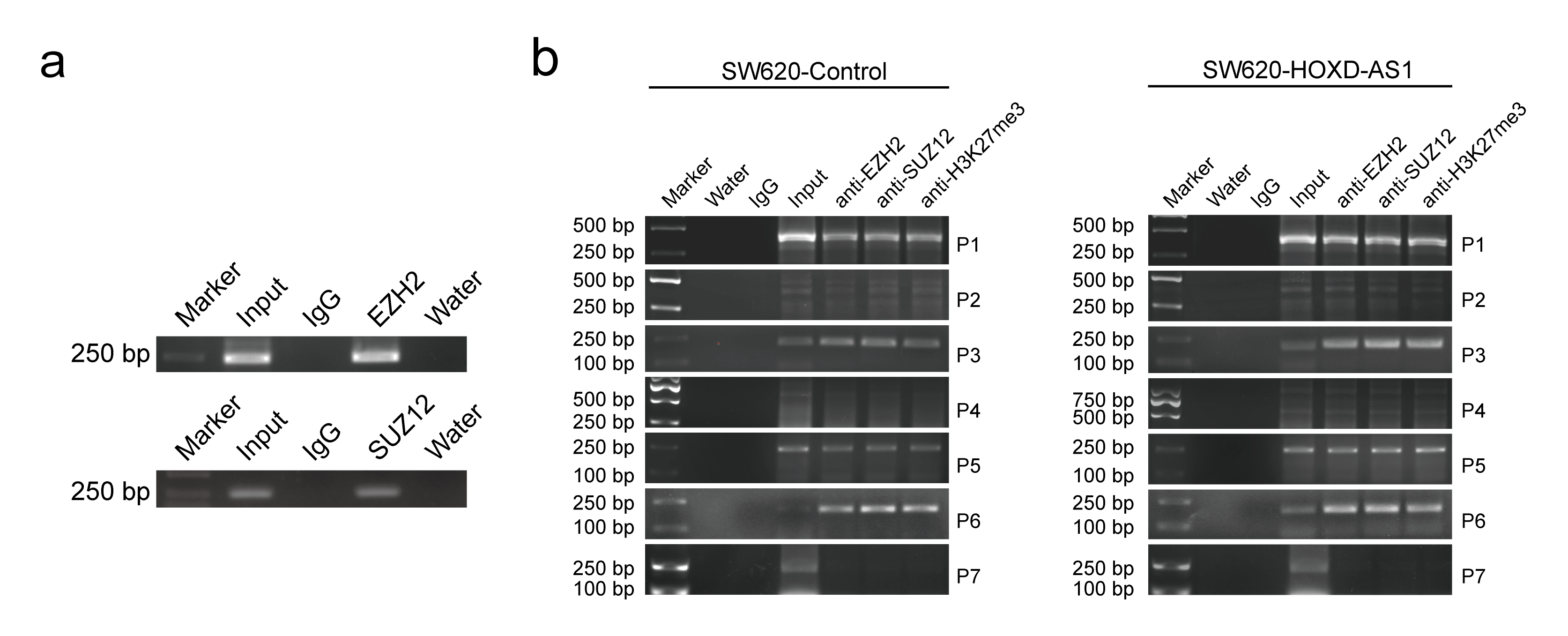

Supplement: Supplementary file 6 — Figure S4. HOXD-AS1 regulates HOXD3 expression through cooperating with PRC2 complex. (a) RIP assays were performed in SW620 cells using anti-SUZ12- antibodies, anti-EZH2- antibodies or nonspecific IgG antibodies respectively. Real-time PCR was performed to determine amount of RNA associated with SUZ12, EZH2 or IgG compared with the input control. (b) ChIP assays were performed in HOXD-AS1 overexpressed(SW620-HOXD-AS1)and control cells using anti-EZH2, anti-SUZ12, anti-H3K27me3 or IgG antibodies respectively. The ChIP products were amplified by real-time PCR. (TIF 3699 kb) [file 12943_2019_955_MOESM6_ESM.tif]

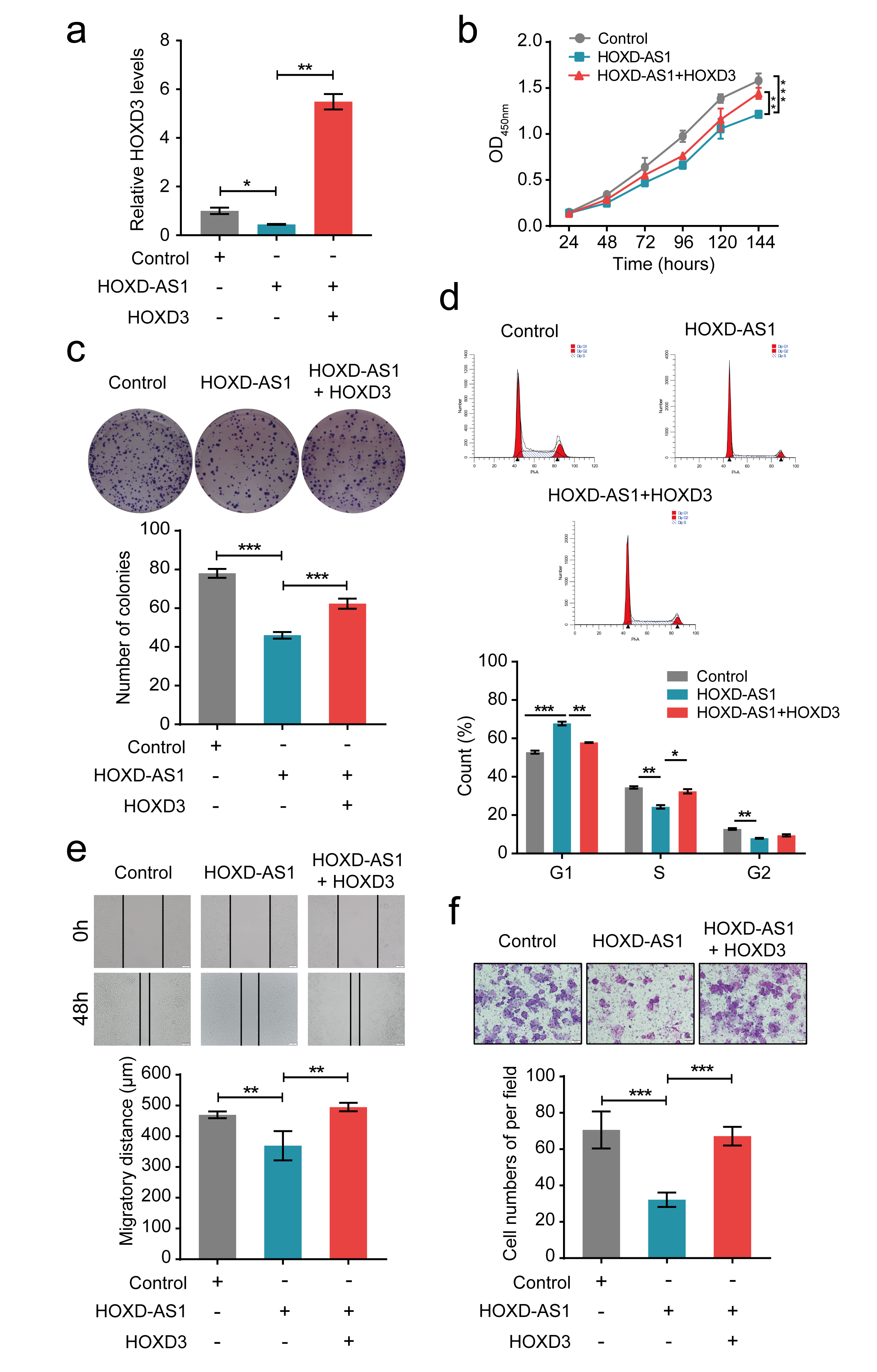

Supplement: Supplementary file 7 — Figure S5. HOXD3 is required for the HOXD-AS1-mediated progress of CRC in vitro. (a) Real-time PCR analysis of HOXD3 expression in SW620-HOXD-AS1, SW620-HOXD-AS1 + HOXD3 and control cells. HOXD3 level was normalized to GAPDH expression. (b) CCK-8 assay, (c) colony formation assay and (d) cell cycle progression assay were performed to determine the cell proliferative ability. (e) Wound healing assay and (f) Transwell assay were used to examine the migratory and invasive abilities of CRC cells. For a-f, the date were expressed as mean ± SD in three independent experiments. *P < 0.05, **P < 0.01, ***P < 0.001. (TIF 5471 kb) [file 12943_2019_955_MOESM7_ESM.tif]

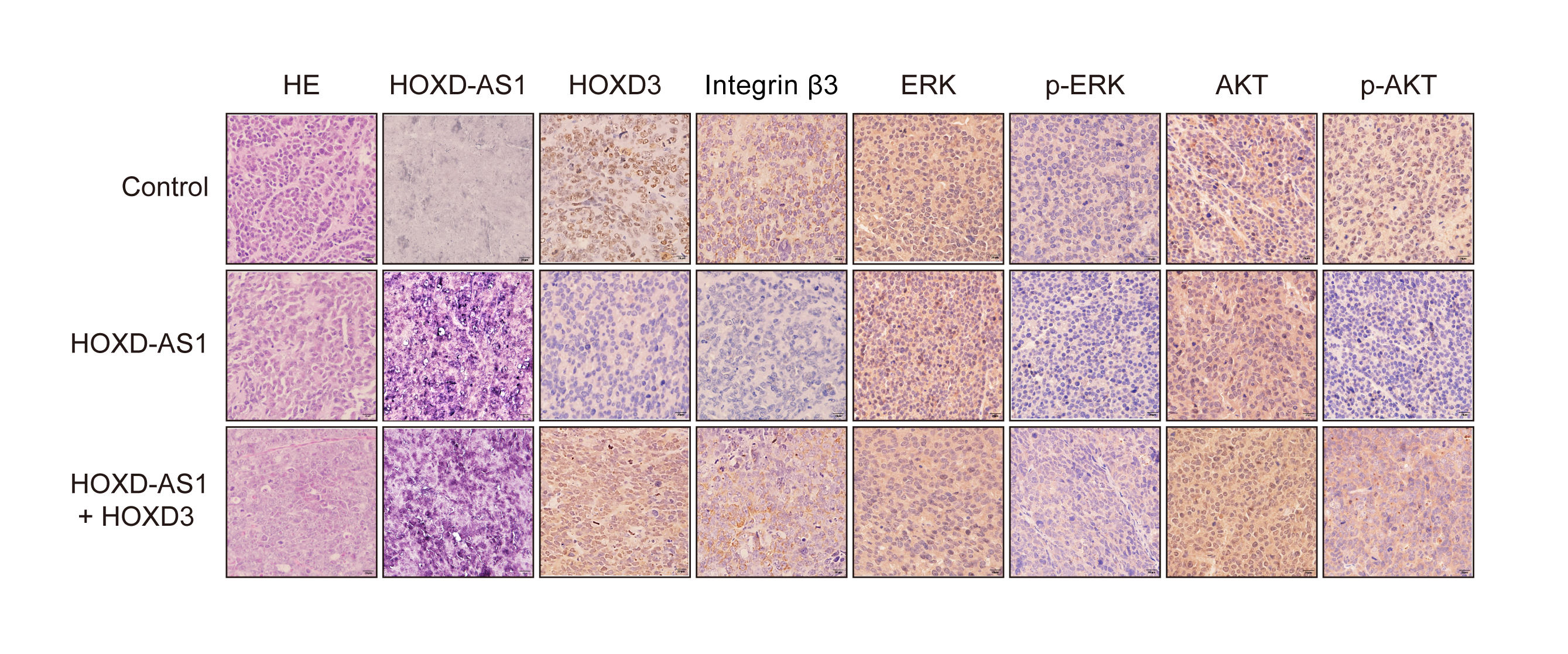

Supplement: Supplementary file 8 — Figure S6. Examine the expression of HOXD3 and Integrin β3/MAPK/AKT signaling in xenografts by IHC assays. (TIF 9353 kb) [file 12943_2019_955_MOESM8_ESM.tif]

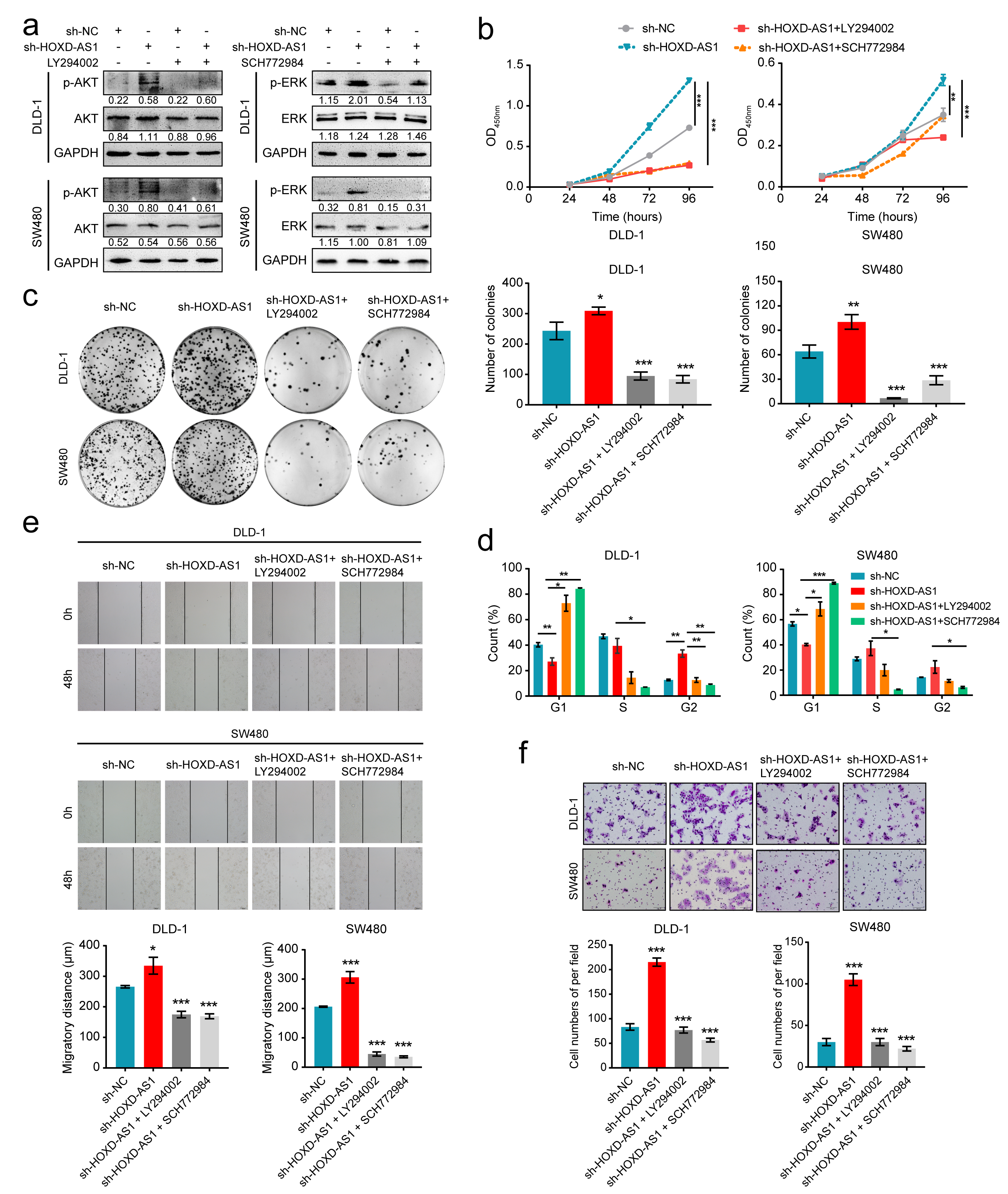

Supplement: Supplementary file 9 — Figure S7. HOXD-AS1 regulates CRC progression through the MAPK/AKT signaling pathways. (a) Detected AKT, p-AKT, ERK, p-ERK protein level in SW480 and DLD-1 cells after being treated with inhibitor of ERK (SCH772984) or AKT (LY294002), respectively. CCK-8 assay (b) colony formation assay (c) and cell cycle progression assay (d) were performed to determine the cell proliferative ability of CRC cells. (e) Wound healing assay and (f) Transwell assay were used to examine the migratory and invasive abilities of CRC cells. For b-f, the date were expressed as mean ± SD in three independent experiments. *P < 0.05, **P < 0.01, ***P < 0.001. (TIF 9210 kb) [file 12943_2019_955_MOESM9_ESM.tif]
